# Tree-aggregated predictive modeling of microbiome data

**DOI:** 10.1038/s41598-021-93645-3

**Published:** 2021-07-15

**Authors:** Jacob Bien, Xiaohan Yan, Léo Simpson, Christian L. Müller

**Affiliations:** 1 Department of Data Sciences and Operations, University of Southern California, Los Angeles, CA USA; 2 Microsoft Azure, Redmond, WA USA; 3 Technische Universität München, Munich, Germany; 4 Institute of Computational Biology, Helmholtz Zentrum München, Munich, Germany; 5 Department of Statistics, Ludwig-Maximilians-Universität München, Munich, Germany; 6 Center for Computational Mathematics, Flatiron Institute, Simons Foundation, New York, NY USA

**Keywords:** Microbial communities, Statistical methods, Software

## Abstract

Modern high-throughput sequencing technologies provide low-cost microbiome survey data across all habitats of life at unprecedented scale. At the most granular level, the primary data consist of sparse counts of amplicon sequence variants or operational taxonomic units that are associated with taxonomic and phylogenetic group information. In this contribution, we leverage the hierarchical structure of amplicon data and propose a data-driven and scalable tree-guided aggregation framework to associate microbial subcompositions with response variables of interest. The excess number of zero or low count measurements at the read level forces traditional microbiome data analysis workflows to remove rare sequencing variants or group them by a fixed taxonomic rank, such as genus or phylum, or by phylogenetic similarity. By contrast, our framework, which we call trac (tree-aggregation of compositional data), learns data-adaptive taxon aggregation levels for predictive modeling, greatly reducing the need for user-defined aggregation in preprocessing while simultaneously integrating seamlessly into the compositional data analysis framework. We illustrate the versatility of our framework in the context of large-scale regression problems in human gut, soil, and marine microbial ecosystems. We posit that the inferred aggregation levels provide highly interpretable taxon groupings that can help microbiome researchers gain insights into the structure and functioning of the underlying ecosystem of interest.

## Introduction

Microbial communities populate all major environments on earth and significantly contribute to the total planetary biomass. Current estimates suggest that a typical human-associated microbiome consists of $\sim 10^{13}$ bacteria^1^ and that marine bacteria and protists contribute to as much as 70% of the total marine biomass^2^. Recent advances in modern targeted amplicon and metagenomic sequencing technologies provide a cost effective means to get a glimpse into the complexity of natural microbial communities, ranging from marine and soil to host-associated ecosystems^3–5^. However, relating these large-scale observational microbial sequencing surveys to the structure and functioning of microbial ecosystems and the environments they inhabit has remained a formidable scientific challenge.

Microbiome amplicon surveys typically comprise sparse read counts of marker gene sequences, such as 16S rRNA, 18S rRNA, or internal transcribed spacer (ITS) regions. At the most granular level, the data are summarized in count or relative abundance tables of operational taxonomic units (OTUs) at a prescribed sequence similarity level or denoised amplicon sequence variants (ASVs)^6^. The special nature of the marker genes enables taxonomic classification^7–10^ and phylogenetic tree estimation^11^, thus allowing a natural hierarchical grouping of taxa. This grouping information plays an essential role in standard microbiome analysis workflows. For example, a typical amplicon data preprocessing step uses the grouping information for count aggregation where OTU or ASV counts are pooled together at a higher taxonomic rank (e.g., the genus level) or according to phylogenetic similarity^12–16^. This approach reduces the dimensionality of the data set and avoids dealing with the excess number of zero or low count measurements at the OTU or ASV level. In addition, rare sequence variants with incomplete taxonomic annotation are often simply removed from the sample.

This common practice of aggregating to a fixed taxonomic or phylogenetic level and then removing rare variants comes with several statistical and epistemological drawbacks. A major limitation of the fixed-level approach to aggregation is that it forces a tradeoff between, on the one hand, using low-level taxa that are too rare to be informative (requiring throwing out many of them) and, on the other hand, aggregating to taxa that are at such a high level in the tree that one has lost much of the granularity in the original data. Aggregation to a fixed level attempts to impose an unrealistic “one-size-fits-all” mentality onto a complex, highly diverse system with dynamics that likely vary appreciably across the range of species represented. A fundamental premise of this work is that the decision of how to aggregate should not be made globally across an entire microbiome data set a priori but rather be integrated into the particular statistical analysis being performed. Many factors, both biological and technical, contribute to the question of how one should aggregate: biological factors include the characteristics of the ecosystem under study and the nature of the scientific question; technical aspects include the abundance of different taxa, the available quality of the sequencing data—including sequencing technology, sample sequencing depth, and sample size—all of which may affect the ability to distinguish nearby taxa.

Another important factor when considering the practice of aggregating counts is that standard amplicon counts only carry relative (or “compositional”) information about the microbial abundances and thus require dedicated statistical treatment. When working with relative abundance data, the authors in^17–19^ posit that counts should be combined with geometric averages rather than arithmetic averages. The common practice of performing arithmetic aggregation of read counts to some fixed level before switching over to the geometric-average-based compositional data analysis workflow is unsatisfactory since the “optimal” level for fixed aggregation is likely data-dependent, and the mixed use of different averaging operations complicates interpretation of the results.

**Figure 1:**
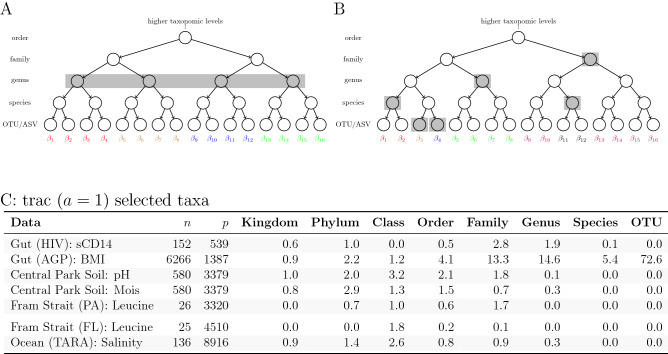
Illustration of fixed level and trac-based taxon aggregation. The trees represent the available taxonomic grouping of 16 *base level* taxa at the leaves (here OTU or ASV). **(A)** Arithmetic aggregation of OTUs/ASVs to a fixed level (genus rank). All taxon base level counts are summed up to the respective parent genus. **(B)** trac’s flexible tree-based aggregation in which the choice of what level to aggregate to can vary across the tree (e.g., two OTUs/ASVs, two species, one genus, and one family). The aggregation is based on the *geometric* mean of OTU/ASV counts and determined in a data-adaptive fashion with the goal of optimizing to the particular prediction task. **(C)** Summary statistics of standard trac-inferred aggregation levels on all seven regression tasks. The Data column denotes the respective regression scenario (study name and outcome of interest), *n* the number of samples, and *p* the number of *base level* taxa (OTUs) in the data. The values in the taxonomic rank columns (Kingdom, Phylum, etc.) indicate the average number of taxa selected on that level by trac  in the respective regression task. Averages are taken over ten random training/out-of-sample test data splits.

To address these concerns, we propose a flexible, data-adaptive approach to tree-based aggregation that fully integrates aggregation into a statistical predictive model rather than relegating aggregation to preprocessing. Given a user-defined taxon *base level* (by default, the OTU/ASV level), our method trac (tree-aggregation of compositional data) learns dataset-specific taxon aggregation levels that are optimized for *predictive regression* modeling, thus making user-defined aggregation obsolete. Using OTU/ASVs as *base level*, Fig. 1A illustrates the typical aggregation-to-genus level approach whereas Fig. 1B shows the prediction-dependent trac approach. The trac method is designed to mesh seamlessly with the compositional data analysis framework by combining log-contrast regression^20^ with tree-guided regularization, recently put forward in^21^. Thanks to the convexity of the underlying penalized estimation problem, trac can deliver interpretable aggregated solutions to large-scale microbiome regression problems in a fast and reproducible manner.

We demonstrate the versatility of our framework by analyzing seven representative regression problems on five datasets covering human gut, soil, and marine microbial ecosystems. Figure 1C summarizes the seven scenarios in terms of size of the microbial datasets and the average number of taxonomic aggregation levels selected by trac-inferred in the respective regression tasks. For instance, for the prediction of sCD14 concentrations (an immune marker in HIV patients) from gut microbiome data, trac selects, on average (over ten random training/test experiments), more taxa at the family level than any other taxonomic level, while it selects no taxa at the class or OTU level. By contrast, for the prediction of pH in the Central Park Soil data, class level taxa are selected more on average than any other level. This highlights the considerable departure from a typical fixed-level aggregation when prediction is the goal. Furthermore, the variability across the seven scenarios suggests that different amounts of aggregation may be warranted in different data sets.

Our trac framework complements other statistical approaches that make use of the available taxonomic or phylogenetic structure in microbial data analysis. For example,^22^ uses phylogenetic information in the popular unifrac metric to measure distances between microbial compositions. The authors in^23–26^ combine tree information with the idea of “balances” from compositional data analysis^18^ to perform phylogenetically-guided factorization of microbiome data. Others have included the tree structure in linear mixed models^27,28^, use phylogenetic-tree-based regression for detecting evolutionary shifts in trait evolution^29^, and integrate tree-information into regression models for microbiome data^30,31^.

Along with our novel statistical formulation, we offer an easy-to-use and highly scalable software framework for simultaneous taxon aggregation and regression, available in the R package trac at https://github.com/jacobbien/trac. The R package trac also includes a fast solver for standard sparse log-contrast regression^15^ to facilitate comparative analyses and a comprehensive documentation and workflow vignette. All data and scripts to fully reproduce the results in this manuscript are available on Zenodo at https://doi.org/10.5281/zenodo.4734527.

We next introduce trac’s mathematical formulation and discuss the key statistical and computational components of the framework. We also give an overview of the microbial data set collection and the comparative benchmark scenarios. To give a succinct summary of the key aspects of trac modeling on microbiome data, we will present and discuss three of the seven regression scenarios in detail. The other scenarios are available in the Supplementary Material. We conclude the study by highlighting key observations and provide recommendations and viable extensions of the trac framework.

## Materials and methods

### Modeling strategy

Let $y\in \mathbb R^n$ be *n* observations of a variable we wish to predict and let $X\in \mathbb R_+^{n\times p}$ be a matrix with $X_{ij}$ giving the number of (amplicon) reads assigned to taxon *j* in sample *i*. The total number of reads $\sum _j X_{ij}$ in sample *i* is a reflection of the sequencing process and therefore should not be interpreted as providing meaningful information about the biological sample itself. This observation has motivated the adoption of compositional data methods, which ensure that analyses depend only on *relative* abundances. Following the foundational work in^20^, one appropriate model for regression with relative abundance data is the log-contrast model where the outcome of interest is modeled as linear combinations of log-ratios (i.e., log-contrasts) of relative abundance features. For high-dimensional microbiome data, the authors in^15^ propose solving an $\ell _1$-penalized regression estimator that includes a zero-sum constraint on the coefficients, the so-called sparse log-contrast model. Writing $\log (X)$ for the matrix with *ij*th entry $\log (X_{ij})$, their estimator is of the form

1 $$\begin{aligned} \text {minimize}_{\beta \in \mathbb R^p} \quad L\left( y-\log (X)\beta \right) + \lambda \mathcal P(\beta )\ \text { s.t. }{1}_p^T\beta = 0. \end{aligned}$$

Here, $L(r)=(2n)^{-1}\Vert r\Vert ^2$ is the squared error loss and $\mathcal P(\beta ) = \Vert \beta \Vert _1$ is the $\ell _1$ penalty^32^. The zero-sum constraint ensures that this model is equivalent to a log-contrast model^33^ and invariant to sample-specific scaling. To understand the intuition behind the sparse log-contrast model, imagine that $\beta _j$ and $\beta _k$ are the only two nonzero coefficients. In such a case, the zero-sum constraint implies that predictions will be based on only the log-ratio of these two taxa. This can be seen by noting that $\beta _j=-\beta _k$, and so our model’s prediction for observation *i* would be given by the following:

$$\begin{aligned}{}[\log (X)\beta ]_i=\beta _j\log (X_{ij})+\beta _k\log (X_{ik})=\beta _j\log (X_{ij})-\beta _j\log (X_{ik})=\beta _j\log (X_{ij}/X_{ik}). \end{aligned}$$

Thus, using a log has the effect of turning differences into ratios. In addition, the zero-sum constraint provides invariance to sample-specific scaling: Replacing $X$ by $DX$, where $D$ is an arbitrary diagonal matrix, leaves Eq. (1) unchanged:

$$\begin{aligned} {[}\log (DX)\beta ]_i=\sum _{j=1}^p\log (D_{ii}X_{ij})\beta _j=\sum _{j=1}^p\left[ \log (D_{ii})\beta _j+\log (X_{ij})\beta _j\right] =0+[\log (X)\beta ]_i. \end{aligned}$$

The choice of the $\ell _1$ penalty was motivated in^15^ by the high dimensionality of microbiome data and the desire for parsimonious predictive models. However, such a penalty is not well-suited to situations in which large numbers of features are highly rare^21^, a well-known feature of amplicon data. A common remedy, also adopted in^15^, is to aggregate taxa at the base level, e.g., OTUs or ASVs, to the genus level and then to screen out all but the most abundant genera. Figure 1A depicts this standard practice: taxonomic (or phylogenetic) information in the form of a tree $\mathcal T$ is used to aggregate data, usually in an arithmetic manner (i.e. by summing), to a *fixed level* of the tree.

Our goal is to make aggregation more flexible (as illustrated in Fig. 1B), to allow the prediction task to inform the decision of how to aggregate, and to do so in a manner that is consistent with the log-contrast framework introduced above. A key insight is that aggregating features can be equivalently expressed as setting elements of $\beta$ equal to each other. For example, suppose we partition the *p* base level taxa into *K* groups $G_1,\ldots , G_K$ and demand that $\beta$ be constant within each group. Doing so yields *K* aggregated features. If all of the $\beta _j$ in group $G_k$ are equal to some common value $\gamma _k$, then

$$\begin{aligned} \sum _j\beta _j\log (X_{ij}) = \sum _{k=1}^K\gamma _k\left( \sum _{j\in G_k}\log (X_{ij})\right) =\sum _{k=1}^K\gamma _k|G_k|\cdot \log \left[ (\prod _{j\in G_k}X_{ij})^{1/{G_k}}\right] . \end{aligned}$$

Thus, we are left with a linear model with *K* aggregated features, each being proportional to the log of the geometric mean of the base level taxa counts.

Associating the elements of $\beta$ with the leaves of $\mathcal T$, the above insight tells us that if our estimate of $\beta$ is constant within subtrees of $\mathcal T$, then that corresponds to a regression model with tree-aggregated features. In particular, each subtree with constant $\beta$-values will correspond to a feature, which is the log of the geometric mean of the counts within that subtree. The trac estimator uses a convex, tree-based penalty $\mathcal P_\mathcal T(\beta )$ for the penalty in Eq. (1) that is specially designed to promote $\beta$ to have this structure that is based on subtrees of $\mathcal T$. The mathematical form of $\mathcal P_\mathcal T(\beta )$ is given in Supplementary Material B. There, we show that the trac estimator reduces to solving the optimization problem:

2 $$\begin{aligned} \text {minimize}_{\alpha \in \mathbb R^{|\mathcal T|-1}} \quad L\left( y-\log (\text {geom}(X;\mathcal T))\alpha \right) + \lambda \sum _{u\in \mathcal T-\{r\}}w_u|\alpha _u| \text { s.t. }{{1}_{|\mathcal T|-1}}^T \alpha = 0, \end{aligned}$$

where $\text {geom}(X;\mathcal T)\in \mathbb R^{n\times (|\mathcal T|-1)}$ is a matrix where each column corresponds to a non-root node of $\mathcal T$ and consists of the geometric mean of all base level taxa counts within the subtree rooted at *u*. Comparing this form of the trac optimization problem to Eq. (1) reveals an alternate perspective: trac can be interpreted as being like a sparse log-contrast model but instead of the features corresponding to base level taxa, they correspond to the geometric means of non-root taxa in $\mathcal T$ (i.e., *X* is replaced by $\text {geom}(X;\mathcal T)$). This also facilitates model interpretability since we can directly combine positive and negative predictors into pairs of log-ratio predictors. For example, if taxa $\alpha _u>0$ and $\alpha _v<0$ are the only nonzero coefficients, then our predictions would be based on

$$\begin{aligned} \log \left[ \frac{\text {geom}(X;\mathcal T)_u}{\text {geom}(X;\mathcal T)_v}\right] . \end{aligned}$$

The particular choice of penalty is a weighted $\ell _1$-norm. While the trac package allows the user to specify general choices of weights $w_u>0$, a convenient and interpretable strategy is to set weights to be an inverse power of the number of leaves in the subtree rooted at *u*, $w_u=|L_u|^{-a}$. The scalar parameter $a \in \mathbb {R}$ controls the overall aggregation strength, with $a=1$ being the default setting in trac. If the user decreases *a*, trac favors aggregations at a lower level of the tree. For *a* sufficiently negative, trac admits solutions equivalent to a sparse log-contrast model without aggregation since only leaves (with $|L_u|=1$) will remain unaffected by the weight scaling. The regularization parameter $\lambda$, on the other hand, is a positive number determining the overall tradeoff between prediction error on the training data and how much aggregation should occur. By varying $\lambda$, we can trace out an entire solution path $\hat{\alpha }(\lambda )$, from highly sparse solutions (large $\lambda$) to more dense solutions involving many taxa (small $\lambda$). This “aggregation path” can itself be a useful exploratory tool in that it provides an ordering of the taxa as they enter the model.

### Computation, model selection, and prediction

Using trac in practice requires the efficient and accurate numerical solution of the convex optimization problem, specified in Eq. (2), across the full aggregation path. We experimented with several numerical schemes and found the path algorithm of^34^ particularly well-suited for this task. The trac R package internally uses the path algorithm implementation from the c-lasso Python package^35^, efficiently solving even high-dimensional trac problems. The trac package also provides a fast implementation of sparse log-contrast regression^15^ for model comparison. The R package reticulate^36^ is instrumental in connecting trac with the underlying Python library. The R packages phyloseq^37^, ggplot2^38^, ape^39^, igraph^40^, and ggtree^41^ are used for operations on tree structures and visualization.

To find a suitable aggregation level along the solution path, we use cross validation (CV) with mean squared error to select the regularization parameter $\lambda \in [\lambda _\text {min},\lambda _\text {max}]$ for all the results presented in this paper. In particular, we perform 5-fold CV with the “one-standard-error rule” (1SE)^42^, which identifies the largest $\lambda$ whose CV error is within one standard error of the minimum CV error. This heuristic purposely favors models that involve fewer taxa and are therefore easier to interpret. (We also use the 1SE rule to select $\lambda$ for the sparse log-contrast model.) The parameter *a* is a user-defined control parameter and not subject to a model selection criterion. Having solved the trac optimization problem and chosen a value of the tuning parameter ($\hat{\lambda }_\text {chosen}$), we can predict the response value at a new sample. Given a new vector of abundances $\tilde{x}\in \mathbb R_+^p$, we predict the response to be

$$\begin{aligned} \hat{y}(\tilde{x})=\sum _{u\in \mathcal T-\{r\}}\hat{\alpha }_u(\hat{\lambda }_\text {chosen})\cdot \log [\text {geom}(\tilde{x};\mathcal T)_u]. \end{aligned}$$

Due to trac’s sparsity penalty, in general only a small number of coefficients will be non-zero, and thus the predictions will depend on only a small number of taxas’ geometric means.

### Data collection

We assembled a collection of five publicly available and previously analyzed datasets, spanning human gut, soil, and marine ecosystems (see also Data column in Fig. 1C). All datasets, except for Tara, consist of 16S rRNA amplicon data of Bacteria and Archaea in the form of OTU count tables, taxonomic classifications, and measured covariates, as provided in the original publications. For ease of interpretability, we leverage the taxonomic tree information rather than phylogeny in our aggregation framework. To investigate potential human host-microbiome interactions, we re-analyze two human gut datasets, one cohort of HIV patients (Gut [HIV]), available in^43^, comprising $p=539$ OTUs and $n=152$ samples, and the other a subset of the American Gut Project data (Gut (AGP))^5^, provided in^44^, comprising $p=1387$ OTUs present in at least $10\%$ of the $n=6266$ samples. To study niche partitioning in terrestrial ecosystems, we use the Central Park soil dataset^45^, as provided by^23^, which consists of $p=3379$ OTUs and $n=580$ samples with a wide range of soil property measurements. For marine microbial ecosystems, we consider a sample collection from the Fram Strait in the North Atlantic^46^, available at https://github.com/edfadeev/Bact-comm-PS85. The data set consists of $n=26$ samples for $p=3320$ OTUs in the particle-associated size class, and $n=25$ samples for $p=4510$ OTUs in the free-living size class. The second marine dataset is the Tara global surface ocean water sample collection^3^, available at http://ocean-microbiome.embl.de/companion.html, which comprises metagenome-derived OTUs (mOTUs). In Tara, each of the $p=8916$ mOTUs considered here is present in at least $10\%$ of the $n=136$ samples. All data and analysis scripts are available in fully reproducible R workflows at https://github.com/jacobbien/trac-reproducible. Since trac  can operate on any taxon base level, we provide all data sets both in the form of the original (m)OTU base level as well as in arithmetically aggregated form on higher-order ranks, i.e., species, genus, family, order, class, and phylum. This facilitates straightforward method comparison across different base level aggregations.

### Method comparison and model quality assessment

To provide a comprehensive model performance evaluation and to highlight the flexibility of the trac modeling framework, we consider the following benchmark scenarios. Firstly, we consider three different regression models. We choose the sparse log-contrast regression model^15^ as the standard baseline of performing regression on compositional data and can be considered as a limiting case of trac. In addition, we consider trac with two different aggregation parameters *a*. The setting $a=1$ is referred to as standard trac. The setting $a=1/2$ is referred to as *weighted* trac and tends to favor aggregations closer to the leaf level. Secondly, to assess the influence of arithmetic aggregation to a fixed level, e.g., the genus level, we compare the performance of all regression models for three different input base levels: OTU, genus, and family level.

To assess how well a log-contrast or trac model generalizes to “unseen” data, we randomly select 2/3 of the samples in each of the considered datasets for model training and selection. On the remaining 1/3 of the samples, we compute out-of-sample test mean squared error as well as the Pearson correlation between model predictions and actual measurements on the test set. While the out-of-sample test error serves as a key quantity to assess model generalizability, we also record overall model sparsity, measured in terms of number of aggregations (or taxa for sparse log-contrast models) in the trained model. Model sparsity serves to measure how “interpretable” a model is. Finally, we repeat all analysis on ten random training/test splits of the data to measure average test error and model sparsity. To ease interpretability, we analyze the trained models derived from split 1 in greater detail throughout the next section and detail the biological significance of the derived regression models.

## Results and discussion

We next highlight key results of the trac framework for three of the seven regression scenarios described above on three different microbiome datasets. The first scenario considers the prediction of an immune marker (soluble sCD14) in HIV patients from microbiome data. In this scenario, we detail the behavior of a typical trac  aggregation path and the model selection process. Furthermore, we compare the performance of trac  models at different taxon base levels (OTU, genus, and family level) and aggregation weights ($a \in \{1/2,1\}$) with standard sparse log-contrast models and analyze the resulting taxa aggregations. In the second scenario, we apply trac  to predict pH concentrations in Central Park soil from microbial abundances and compare the resulting aggregations to known associations of pH and microbial taxa. The last scenario considers salinity prediction in the global ocean from Tara mOTU data. Further trac  prediction scenarios are available in theSupplementary Material, including Body Mass Index (BMI) predictions on the American Gut Project Data, soil moisture prediction in Central Park soil, and primary productivity prediction from marine microbes in two different size fractions in the North Atlantic Fram Strait.

### Immune marker sCD14 prediction in HIV patients

Infection with HIV is often paired with additional acute or chronic inflammation events in the epithelial barrier, leading to disruption of intestinal function and the microbiome. The interplay between HIV infection and the gut microbiome has been posited to be a “two-way street”^47^: HIV-associated mucosal pathogenesis potentially leads to perturbation of the gut microbiome and, in turn, altered microbial compositions could result in ongoing disruption in intestinal homeostasis as well as secondary HIV-associated immune activation and inflammation.

Here, we investigate one aspect of this complex relationship by learning predictive models of immune markers from gut amplicon sequences. While^48^ were among the first to provide evidence that gut microbial *diversity* is a predictor of HIV immune status (as measured by CD4+ cell counts), we consider soluble CD14 (sCD14) measurements in HIV patients as the variable to predict and learn an interpretable regression model from gut microbial amplicon data. sCD14 is a marker of microbial translocation and has been shown to be an independent predictor of mortality in HIV infection^49^.

**Figure 2:**
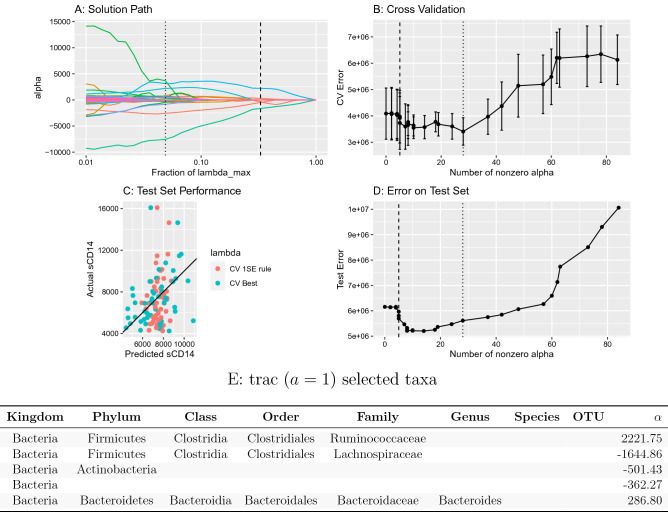
Overview of trac aggregation and model selection with standard weighting $a=1$ on the sCD14 data. **(A)** Varying the trac regularization parameter $\lambda$ produces a solution (aggregation) path. Each colored line corresponds to a distinct taxon, showing its $\alpha$ coefficient value as the tuning parameter $\lambda$ increases. The larger $\lambda$ is, the more coefficients are set to 0, leading to a more parsimonious model. The dotted and dashed vertical lines mark the $\lambda$-values selected by the CV best and 1SE rule, respectively. **(B)** Illustration of the cross-validation (CV) procedure. Mean (and standard error) CV error vs. $\lambda$ path with selected $\lambda$ values at best CV error (dotted vertical line) or with the 1SE rule (dashed vertical line). **(C)** The actual vs. predicted values of sCD14 on the test set (1SE rule in red, CV best in blue). The Pearson correlation of trac predictions on the test set is 0.37 with the CV best solution and 0.23 with the CV 1SE rule, respectively. (**D**) Error on the test set vs. number of selected aggregations. **(E)** The trac model selected with the 1SE rule comprises five taxa across four levels, listed in the bottom table (see Fig. 3A for tree visualization of the aggregations). The column labeled $\alpha$ gives the nonzero coefficient values, which are in the same units as the sCD14 response variable.

Following^43^, we analyze an HIV cohort of $n=152$ patients where sCD14 levels (in pg/ml units) and fecal 16S rRNA amplicon data were measured. Using as base level all available $p=539$ bacterial and archaeal OTUs, we first illustrate the typical trac prediction and model selection outputs with default weight parameter $a=1$ on the first (of overall ten) training/test splits in Fig. 2. In Fig. 2A, we visualize the solution of the $\alpha$ coefficients associated with each aggregation along the regularization path. The vertical lines indicate the aggregations that were selected via cross-validation (CV) with the minimum mean squared error (CV best, dotted line) and one-standard-error rule (1SE rule, dashed line) (see Fig. 2B). On the test data, we highlight the relationship between test prediction performance of the trac models versus the number of inferred aggregations (Fig. 2D). Models between five and 28 aggregations show excellent performance on the test set. trac with the 1SE rule identified a parsimonious model with aggregation to five main taxa (Fig. 2E): the kingdom Bacteria, phylum Actinobacteria and the family Lachnospiraceae are negatively associated, and the family Ruminococcaceae and the genus Bacteroides are positively associated with sCD14 counts, thus resulting in a trac model with three log-contrasts.

From a biological perspective, this trac analysis suggests a strong role of the Ruminococcaceae to Lachnospiraceae family ratio and, to a lesser extent, the Ruminococcaceae to Actinobacteria ratio in predicting mucosal disruption (as measured by sCD14). This follows from observing the large positive $\alpha$ coefficient associated with Ruminococcaceae and the large negative $\alpha$ coefficients associated with Lachnospiraceae and Actinobacteria (and recalling the interpretation of the trac output in terms of log-ratios). The protective or disruptive roles of Ruminococci or Lachnospiraceae in HIV patients is typically considered to be highly species-specific. Moreover, few consistent microbial patterns are known that generalize across studies^50^. For instance,^51^ report high variability and diverging patterns of the differential abundances of individual OTUs belonging to the Ruminococcaceae and Lachnospiraceae family in HIV-negative and HIV-positive participants. Our model posits that, on the family level, consistent effects of these two families are detectable in amplicon data. This also suggests that, with the right aggregation level, a re-analysis of recent HIV-related microbiome data may, indeed, reveal reproducible patterns of different taxon groups in HIV infection.

**Figure 3:**
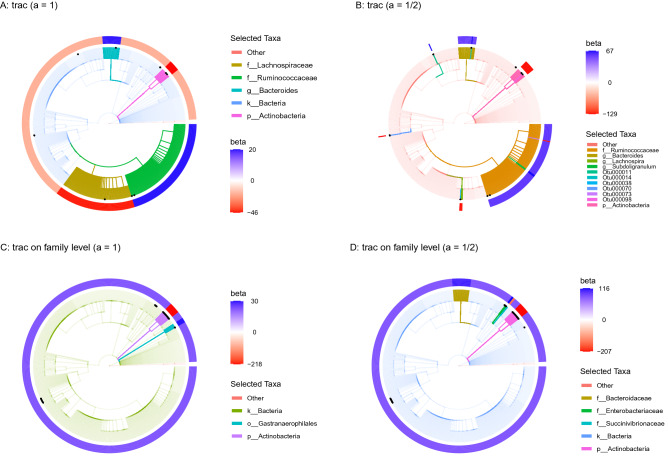
Taxonomic tree visualization of trac aggregations in four selected scenarios using sCD14 data (training/test split 1). Each tree represents the taxonomy of the $p=539$ OTUs. Colored branches highlight the estimated trac taxon aggregations. The black dots mark the selected taxa of the respective sparse log-contrast model. The outer rim represents the value of $\beta$ coefficients in the trac  model from Eq. (1). **(A)** Standard trac ($a=1$) with OTUs as taxon base level selects five aggregations. **(B)** Weighted trac ($a=1/2$) with OTU base level selects eleven aggregations, including six on the OTU level. Four of these OTUs were also selected by the sparse log-contrast model which comprises nine OTUs in total (black dots) (see Suppl. Tables 6 and 7 for the selected coefficients). **(C)** Standard trac ($a=1$) with family base level selects three aggregations. **(D)** Weighted trac ($a=1/2$) with family as taxon base level selects five aggregations, including one family (Enterobacteriacaeae) shared with the sparse log-contrast model when also applied at the family base level (see Suppl. Tables 10 for the six selected families).

To quantify the effect of taxon base level and aggregation weight scaling *a*, we re-analyze the data at OTU, genus, and family base level and compare trac models to sparse log-contrast models at the respective base level. The latter approach thus reflects the default mode of analysis, proposed in^15^, where sparse log-contrast modeling on fixed genus aggregations was performed. Figure 3 visualizes the estimated trac  aggregations ($a \in \{1,1/2\}$) and sparse taxa on the taxonomic tree of the sCD14 data.

Figure 3A,B show the estimated models with OTUs as taxon base level, Fig. 3C,D with family base level. Figure 3A highlights the previously discussed five aggregations from Fig. 2E (Bacteria, Ruminococcaceae, Lachnospiraceae, Actinobacteria, and Bacteroides), found with standard trac ($a=1$), by coloring the respective branches of the corresponding full taxonomic tree. We observe that the selected OTUs of the sparse log-contrast model (highlighted as black dots) cover each of the trac aggregations, including two OTUs in the phylum Actinobacteria, two OTUs in the family Ruminococcaceae, and one OTU in Lachnospiraceae family (see Suppl. Table 7 for the selected coefficients). Figure 3B highlights how weighted trac with $a=1/2$ results in predictive models that can represent a sort of compromise between both standard trac and sparse log-contrast components. For instance, weighted trac still comprises the Ruminococcaceae family, the Actinobacteria phylum, and the Bacteroides genus but also shares four OTUs with the sparse log-contrast model. This exemplifies the flexibility of the trac framework in fine-tuning predictive models to the “right” level of aggregation. We observe a similar but less pronounced effect of the weighting when using aggregated family counts as taxon base level (Fig. 3C,D). The trac models comprise three and five aggregations, respectively, with the Actinobacteria phylum common to both. The sparse log-contrast model comprises six families, three of which are covered by the weighted trac model (two families in the Actinobacteria phylum and the Enterobacteriaceae family).

To compare the different statistical models in terms of interpretability and prediction quality, we report the sparsity level and the out-of-sample prediction errors, averaged over ten different training/test splits, in Table 1. We observe that for the sCD14 data set, standard trac with OTU base levels delivers the sparsest (on average, seven aggregations) and most predictive solution (average test error 6.3e$\,+\,$06), followed by standard trac on the family level (average test error 6.5e$\,+\,$06). The sparse log-contrast model with genus base level has considerably reduced prediction capability (average test error 7.1e$\,+\,$06). On this data set, weighted trac  ($a=1/2$) models show the expected intermediate properties between sparse log-contrast and standard trac solutions.

**Table 1:** Average out-of-sample test errors (rounded average model sparsity in parenthesis) for trac ($a\in\{1, 1/2\}$) and sparse log-contrast models, respectively. Each row considers a different base level (OTU, genus, and family). Each number is averaged over ten different training/test splits of the sCD14 data.

Base level	*p*	trac (a = 1)	trac (a = 1/2)	Sparse log-contrast
OTU	539	6.3e$\,+\,$06 (7)	6.7e$\,+\,$06 (9)	6.8e$\,+\,$06 (8)
Genus	282	6.8e$\,+\,$06 (7)	7.1e$\,+\,$06 (8)	7.1e$\,+\,$06 (9)
Family	112	6.5e$\,+\,$06 (4)	6.5e$\,+\,$06 (5)	6.6e$\,+\,$06 (7)

### Predicting central park soil pH concentration from microbiome data

We next perform trac prediction tasks on environmental rather than host-associated microbiome data. We first consider soil microbial compositions since they are known to vary considerably across spatial scales and are shaped by myriads of biotic and abiotic factors. Using univariate regression models, the authors in^52^ found that soil habitat properties, in particular pH and soil moisture deficit (SMD), can predict overall microbial “phylotype” diversity. For instance, using $n=88$ soil samples from North and South America, the authors in^53^ showed that soil pH concentrations are strongly associated with amplicon sequence compositions, as measured by pairwise unifrac distances. Moreover, they found that soil pH correlated positively with the relative abundances of Actinobacteria and Bacteroidetes phyla, negatively with Acidobacteria, and not at all with Beta/Gammaproteobacteria ratios.

Here, we use trac on the Central Park soil data collection comprising $n=580$ samples and $p=3379$ bacterial and archaeal OTUs^23,45^ to provide a refined analysis of the relationship between soil microbiome and habitat properties. Rather than looking at the univariate correlative pattern between soil properties and phyla, we build multivariate models that take soil pH as the response variable of interest and optimize taxa aggregations using trac and sparse log-contrast models. The predictive analysis for soil moisture is relegated to the Supplementary Materials.

**Figure 4:**
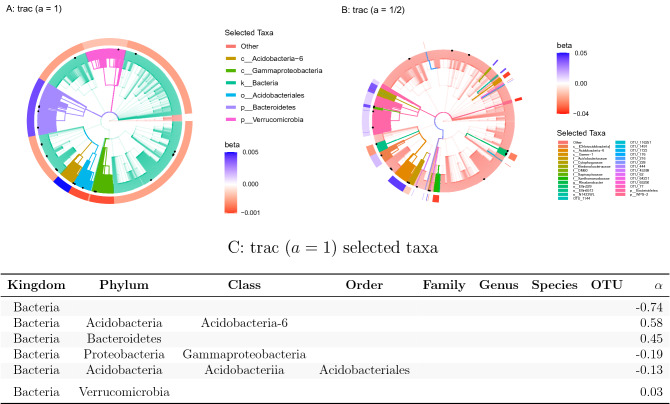
Taxonomic tree visualization of trac aggregations ($a\in\{1, 1/2\}$ using the Central Park soil data (training/test split 1). Each tree represents the taxonomy of the $p=3379$ OTUs. Colored branches highlight the estimated trac taxon aggregations. The black dots mark the selected taxa of the sparse log-contrast model. The outer rim represents the value of $\beta$ coefficients in the trac model from Eq. (1). **(A)** Standard trac ($a=1$) with OTUs as taxon base level selects six aggregations. **(B)** Weighted trac ($a=1/2$) with OTU base level selects 28 aggregations, including 13 on the OTU level. Four of these OTUs are also selected by the sparse log-contrast model which comprises 21 OTUs in total (black dots) (see Suppl. Tables 15 and 16 for the selected coefficients). **(C)** The table lists the $\alpha$ coefficients associated with Eq. (2) for the trac ($a=1$) model corresponding to the tree shown in **(A)**. These values are in the same units as the pH response variable.

For pH prediction, standard trac gives an interpretable model with six aggregated taxonomic groups (see Fig. 4A): the two phyla Bacteroidetes and Verrucomicrobia and the class Acidobacteria-6 were positively associated, whereas the order Acidobacteriales, the class Gammaproteobacteria, and the overall kingdom of Bacteria (compared to Archaea) were negatively associated with pH (see bottom table in Fig. 4). We can thus associate a log-contrast model with three log-ratios of aggregated taxonomic groups with soil pH in Central Park. The overall Pearson correlation between the trac predictive model and the training data was 0.68. On the test data, the model still maintained a high correlation of 0.65. With the standard caveat that regression coefficients do not have the same interpretation (or even necessarily have the same sign) as their univariate counterparts, our model also supports a positive relationship between the Bacteroidetes phylum and pH and gives refined insights into the role of the Acidobacteria phylum. The model posits that the class Acidobacteria-6 is positively related and the order Acidobacteriales (in the Acidobacteriia class) is negatively related with pH. The authors in^23^ observed similar groupings in their phylofactorization of the Central Park soil data. There, the classes Acidobacteria-6 and Acidobacteriia belonged to different “binned phylogenetic units” whose relative abundances increased and decreased along the pH gradient, respectively. Finally, the phylum Verrucomicrobia and the class Gammaproteobacteria, included in our model, have been reported to be highly affected by pH with several species of Gammaproteobacteria particularly abundant in low pH soil^54^.

**Table 2:** Average out-of-sample test errors (rounded average model sparsity in parenthesis) for trac ($a\in\{1, 1/2\}$) and sparse log-contrast models, respectively. Each row represents the results for base level OTU, genus, and family. Each value is averaged over ten different training/test splits of the Central Park soil data.

Base level	*p*	trac (a $=$ 1)	trac (a $=$ 1/2)	Sparse log-contrast
OTU	3379	0.40 (10)	0.39 (18)	0.39 (33)
Genus	2779	0.40 (13)	0.38 (22)	0.39 (26)
Family	1492	0.39 (10)	0.39 (15)	0.40 (29)

In contrast to the sCD14 data analysis, weighted trac  ($a=1/2$) delivers a considerably more fine-grained model with 23 aggregations, including 13 on the OTU level. While the Acidobacteria-6 class is still selected as a whole, weighted trac picks specific OTUs and families in the Gammaproteobacteria class. Similar behavior is observed for the Acidobacteriales order and the Bacteroidetes phylum. Moreover, novel orders, families, genera, and OTUs from the Bacteria kingdom are selected. Four OTUs are shared with the sparse log-contrast model which selects 21 OTUs overall.

To compare the models in terms of interpretability and prediction quality, we report in Table 2 average out-of-sample prediction errors and sparsity levels at three different base levels using ten different training/test splits. We observe that for the Central Park soil data set, standard trac with OTU base levels delivers the sparsest solutions (on average, ten aggregations), followed by weighted trac on the family level (on average, 15 aggregations). The sparse log-contrast models deliver the densest models (26–33, on average). All models are comparable in terms of out-of-sample test error (0.38–0.40).

### Global predictive model of ocean salinity from Tara data

Integrative marine data collection efforts such as Tara Oceans^55^ or the Simons CMAP (https://simonscmap.com) provide the means to investigate ocean ecosystems on a global scale. Using Tara’s environmental and microbial survey of ocean surface water^3^, we next illustrate how trac can be used to globally connect environmental covariates and marine microbiome data. As an example, we learn global predictive models of ocean salinity from $n=136$ samples and $p=8916$ miTAG OTUs^56^. Even though salinity is thought to be an important environmental factor in marine microbial ecosystems, existing studies have investigated the connection between the microbiome and salinity gradients mainly on a local marine scale, in particular estuaries.

**Figure 5:**
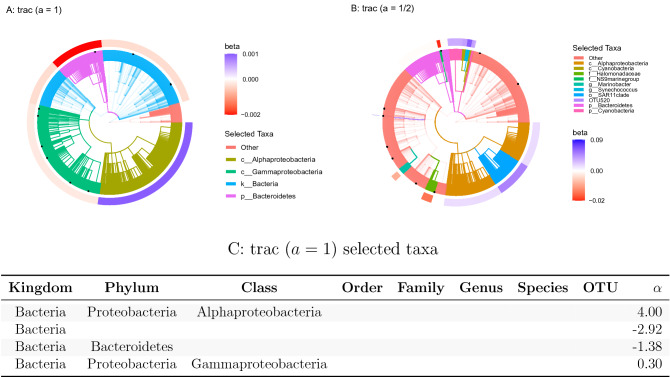
Taxonomic tree visualization of trac aggregations (OTUs as taxon base level, $a\in\{1, 1/2\}$ for salinity prediction using Tara data (training/test split 1). Each tree represents the taxonomy of the $p=8916$ miTAG OTUs. Colored branches highlight the estimated trac taxon aggregations. The black dots mark the selected taxa of the sparse log-contrast model. The outer rim represents the value of $\beta$ coefficients in the trac model from Eq. (1). **(A)** Standard trac ($a=1$) selects four aggregations on the kingdom, phylum, and class level. **(B)** Weighted trac ($a=1/2$) selects ten aggregations across all taxonomic ranks, including a single OTU (OTU520). This OTU is also selected by the sparse log-contrast model which comprises nine OTUs in total (black dots) (see Suppl. Table 18 for the selected coefficients). Both trac models select the phylum Bacteroidetes and the Alphaproteobacteria class. **(C)** The table lists the $\alpha$ coefficients associated with Eq. (2) for the trac ($a=1$) model corresponding to the tree shown in **( A)**. These values are in the same units as the salinity response variable.

Standard trac ($a=1$) identifies four taxonomic aggregations (see Fig. 5A), the kingdom Bacteria and the phylum Bacteroidetes being negatively associated and the class Alphaproteobacteria being strongly positively and Gammaproteobacteria being moderately positively associated with marine salinity.

Consistent with this trac model, a marked increase of Alphaproteobacteria with increasing salinity was observed in several estuary studies^57,58^. In a global marine microbiome meta-analysis^59^, Spearman rank correlations between relative abundances of microbial clades and several physicochemical water properties, including salinity, were reported, showing four out of five orders in the Bacteroidetes phylum to be negatively correlated with salinity. However, three out of four orders belonging to Gammaproteobacteria were negatively correlated with salinity, suggesting that the standard trac model does not universally agree with standard univariate assessments. However, as shown in Fig. 5B, weighted trac ($a=1/2$) reveals a more fine-grained taxon aggregation, selecting the Halomonadaceae family and the Marinobacter genus in the phylum Gammaproteobacteria with negative $\alpha$ coefficients and a Gammaproteobacteria OTU (OTU 520, order E01-9C-26 marine group) with positive $\alpha$ coefficients, respectively (see also Supplementary Table 23). Likewise, out of the nine OTUs selected by the sparse log-contrast model (black dots in Fig. 5A,B), four out of six selected Gammaproteobacteria OTUs have negative coefficients (including OTU 520), and two OTUs have positive coefficients.

In terms of model performance, the standard trac model shows good global predictive capabilities with an out-of-sample test error of 1.99 (on training/test split 1). We observe, however, that high salinity outliers located in the Red Sea (Coastal Biome) and the Mediterranean Sea (Westerlies Biome) and a low salinity outlier (far eastern Pacific fresh pool south of Panama) are not well captured by the model (see Supplementary Figure 5 for a scatter plot of measured vs. predicted salinity). Weighted trac ($a=1/2$) and the sparse log-contrast models outperform standard trac on the salinity prediction task with an out-of-sample test error (on split 1) of 1.94 and 1.52, respectively.

This boost in prediction quality is further confirmed by the average out-of-sample prediction errors across all ten training/test splits and three base levels (see Table 3). Sparse log-contrast models on the OTU and Genus base level perform best (average test error 1.3 and 1.4, respectively), followed by weighted trac  on Genus level (1.5). However, standard trac models are considerably sparser (six to seven aggregations) compared to log-contrast models (13–24 taxa). Weighted trac models represent a good trade-off between predictability and interpretability, selecting ten to fourteen taxa, on average.

**Table 3:** Average out-of-sample test errors (rounded average model sparsity in parenthesis) for trac ($a\in\{1, 1/2\}$) and sparse log-contrast models, respectively. Each row represents the results for base level OTU, genus, and family and the corresponding dimensionality of the base level. Each value is averaged over ten different training/test splits of the Tara data.

Base level	*p*	trac (a $=$ 1)	trac (a $=$ 1/2)	Sparse log-contrast
OTU	8916	2.1 (7)	1.8 (14)	1.3 (24)
Genus	4220	2.0 (7)	1.5 (14)	1.4 (34)
Family	1869	2.1 (6)	1.7 (10)	1.6 (13)

## Conclusions

Finding predictive and interpretable relationships between microbial amplicon sequencing data and ecological, environmental, or host-associated covariates of interest is a cornerstone of exploratory data analysis in microbial biogeography and ecology. To this end, we have introduced trac, a scalable tree-aggregation regression framework for compositional amplicon data. The framework leverages the hierarchical nature of microbial sequencing data to learn parsimonious log-ratios of microbial compositions along the taxonomic or phylogenetic tree that best predict continuous environmental or host-associated response variables. The trac method is applicable to any user-defined taxon base level as input, e.g., ASV/OTU, genus, or family level, and includes a scalar tuning parameter *a* that allows control of the overall aggregation granularity. As shown above, this allows seamless testing of a continuum of models to a data set of interest, with prior approaches to sparse log-contrast modeling modeling as special limit cases^15,43,60^. The framework, available in the R package trac and Python^35^, shares similarities with ideas from tree-guided, *balance* modeling of compositional data^18,23,24^, albeit with a stronger focus on finding *predictive* relationships and emphasis on fast computation thanks to the convexity of the formulation and the underlying efficient path algorithm.

Our comprehensive benchmarks and comparative analysis on host-associated and environmental microbiome data revealed several notable observations. Firstly, across almost all tested taxon base levels and methods, standard trac ($a=1$) resulted in the most parsimonious models and revealed data-specific taxon aggregations comprising all taxonomic orders. This facilitated straightforward model interpretability despite the high-dimensionality of the data. For instance, on the sCD14 data, the standard trac model with OTU base level asserted a particularly strong predictive role of the Ruminococcaceae/Lachnospiraceae family ratio for sCD14, thus generating a testable biological hypothesis. Likewise, trac analysis on environmental microbiomes in soil and marine habitats consistently provided parsimonious taxonomic aggregations for predicting covariates of interest. For instance, Alpha- and Gammaproteobacteria/Bacteroidetes ratios well-aligned with sea surface water salinity on a global scale, reminiscent of the ubiquitous Firmicutes/Bacteroidetes ratio in the context of the gut microbiome and obesity^61,62^.

Secondly, arithmetic aggregation of OTUs to a higher taxonomic base level prior to trac  or sparse log-contrast modeling did not result in significant predictive performance gains. In fact, using OTUs as base level, at least one of the three statistical methods showed superior test error performance while maintaining a high level of sparsity. These results suggest that a user may safely choose the highest level of resolution of the data (e.g., mOTUs, OTUs, or ASVs) in (weighted) trac models without sacrificing prediction performance.

Thirdly, while standard trac models always showed good predictive performance on out-of-sample test data, our comparative and average analysis indicated that weighted trac and sparse log-contrast models can outperform the parsimonious trac models in terms of test error, particularly on environmental microbiome data. For instance, on Central Park soil data, we observed moderate performance gains using weighted trac, and on marine data (see Extended Results in the Supplementary Materials for the Fram Strait dataset), sparse log-contrast models showed, on average, the best predictive performance. These results add a valuable piece of information to the ongoing debate about the usefulness of incorporating phylogenetic or taxonomic information into statistical modeling. For example, the authors in^63^ convincingly argue that incorporating such information provides no gains in microbial differential abundance testing scenarios.

We posit that, in the context of statistical regression, full comparative trac analyses like the ones presented here, can immediately determine in a concrete and objective way whether phylogenetic or taxonomic information is useful for a particular prediction task on the data set of interest.

The trac framework naturally lends itself to several methodological extensions that are easy to implement and may prove valuable in microbiome research. Firstly, as apparent in the gut microbiome context, inclusion of additional factors such as diet and life style would likely improve prediction performance. This can be addressed by combining trac with standard (sparse) linear regression to allow the incorporation of (non-compositional) covariates into the statistical model (see, e.g.,^64^). Secondly, while we focused on predictive regression modeling of continuous outcomes, it is straightforward to adopt our framework to classification tasks when binary outcomes, such as, e.g., case vs. control group, or healthy vs. sick participants, are to be predicted. For instance, using the (Huberized) square hinge loss (see, e.g.,^65^) as objective function $L(\cdot )$ in Eq. (2) would provide an ideal means to handle binary responses while simultaneously enabling the use of efficient path algorithms (see^35^ and references therein). Thirdly, due to the compositional nature of current amplicon data, we presented trac in the common framework of log-contrast modeling. However, alternative forms of tree aggregations over compositions are possible, for instance, by directly using the relative abundances as features rather than log-transformed quantities. Tree aggregations would then amount to grouped relative abundance *differences* and not log-ratios, thus resulting in a different interpretation of the estimated model features.

In summary, we believe that our methodology and its implementation in the R package trac, together with the presented reproducible application workflows, provide a valuable blueprint for future data-adaptive aggregation and regression modeling for microbial biomarker discovery, biogeography, and ecology research. This, in turn, may contribute to the generation of new interpretable and testable hypotheses about host-microbiome interactions and the general factors that shape microbial ecosystems in their natural habitats.

## Supplementary Information

Supplementary Information.
