# Supplementary material for: Tree-aggregated predictive modeling of microbiome data
Source: Sci Rep. 2021 Jul 15;11:14505. doi: 10.1038/s41598-021-93645-3 (PMC8282688; doi:10.1038/s41598-021-93645-3)
Supplement: Supplementary file 1 — Supplementary Information. [file 41598_2021_93645_MOESM1_ESM.pdf]

Tree-Aggregated Predictive Modeling of Microbiome Data - Supplementary Material

Jacob Bien [1], Xiaohan Yan [2], Léo Simpson [3,4], and Christian L. Müller[4,5,6,*]

[1]Department of Data Sciences and Operations, University of Southern California, CA, USA

[2]Microsoft Azure, Redmond, WA, USA

[3]Technische Universität München, Germany

[4]Institute of Computational Biology, Helmholtz Zentrum München, Germany

[5]Department of Statistics, Ludwig-Maximilians-Universität München, Germany

[6]Center for Computational Mathematics, Flatiron Institute, Simons Foundation, NY, USA
*correspondence to: cmueller@flatironinstitute.org

A Data and Code availability

The data and code for fully reproducing all results presented in this manuscript are available at Zenodo at `https://doi.org/10.5281/zenodo.4734527`. The simulation code has been tested on R version 4.0. The `trac` R package is available at `https://github.com/jacobbien/trac`. A vignette describing key functionalities of the package and an archetypical workflow are available at `https://jacobbien.github.io/trac/articles/trac-example.html`. The `c-lasso` Python package [1] is available at `https://github.com/Leo-Simpson/c-lasso` and can be installed via `pip`.

B Derivation of Optimization Problem

We design a convex tree-based penalty $\mathcal{P}_{\mathcal{T}}(\beta)$ that promotes β to be constant along branches of $\mathcal{T}$. We encode $\mathcal{T}$ through a binary matrix $A \in \{0,1\}^{p\times(|\mathcal{T}|-1)}$ indicating whether feature j is a leaf of each non-root node $u \in \mathcal{T} - \{r\}$, that is $A_{ju} = 1\{j \in \mathcal{L}(u)\}$ where $\mathcal{L}(u)$ is the set of leaves that descend from u. In particular, we take

$$\mathcal{P}_{\mathcal{T}}(\beta) = \min_{\gamma\in\mathbb{R}^{|\mathcal{T}|-1}} \{\|\gamma\|_1 \quad \text{s.t.} \quad \beta = A\gamma\}.$$

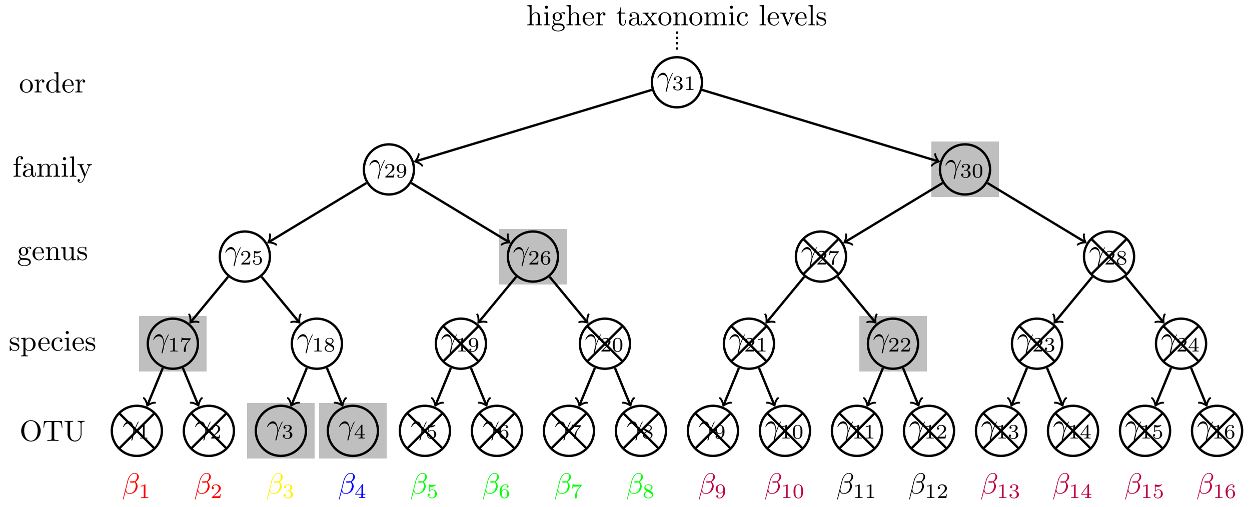

Figure 1: Schematic of the tree aggregation process.

Figure 1 shows a schematic of the tree aggregation idea. The vector $\gamma \in \mathbb{R}^{|\mathcal{T}|-1}$ can be thought of as a latent parameter vector with an entry associated with each node of the tree (see Figure 1). We associate a β_j to each leaf of $\mathcal{T}$, and the constraint $\beta = A\gamma$ expresses a particular relationship between these, namely that each coefficient β_j is the sum of the γ_u for which $j \in \mathcal{L}(u)$ (i.e., each β_j is the sum of its ancestor γ-values in the tree). This relationship implies that when all the γ-values in a subtree are zero (denoted by crossed out nodes in the figure), then all the β coefficients within the subtree are equal. Thus, the sparsity inducing ℓ_1-norm on γ in $\mathcal{P}_{\mathcal{T}}(\beta)$ induces β to tend to be constant within subtrees of $\mathcal{T}$. Using this penalty in Eq. (1) in the main paper leads to the `trac` method, which is computed by solving,

$$\text{minimize}_{\beta\in\mathbb{R}^p,\gamma\in\mathbb{R}^{|\mathcal{T}|-1}} \quad L\left(y - \log(X)\beta\right) + \lambda\|\gamma\|_1 \text{ s.t. } 1_p^T\beta = 0, \quad \beta = A\gamma. \tag{1}$$

This estimator is built on the tree-based aggregation penalty in [2], developed for general situations in which features are rare and a tree relating the features is available. In their setting, features are not compositional, so they do not introduce a sum-to-zero constraint or take the log of the features. The `trac` problem can be written more simply, entirely in terms of γ, as

$$\text{minimize}_{\gamma\in\mathbb{R}^{|\mathcal{T}|-1}} \quad L\left(y - \log(X)A\gamma\right) + \lambda\|\gamma\|_1 \text{ s.t. } 1_p^T A\gamma = 0.$$

The $n \times (|\mathcal{T}|-1)$ matrix $\log(X)A$ has the sum of the log counts of each of the $|\mathcal{T}|-1$ subtrees of $\mathcal{T}$ (excluding $\mathcal{T}$ itself). Changing variables to $\alpha_u = \gamma_u \cdot |\mathcal{L}(u)|$ and using properties of logarithms establishes the equivalence with problem Eq. (2) in the main paper.

C Extended Results

We provide extended results, including an in-depth analysis of `trac` prediction of BMI from American Gut Project data, moisture prediction in Central Park soil, and leucine prediction in the Fram Strait.

Immune marker sCD14 prediction in HIV patients

For the sCD14 data, we provide coefficient tables learned by `trac` ($a = 1$), `trac` ($a = 1/2$), and the sparse log-contrast model on the first random train-test data split (of ten) in Section D. This complements the tree visualizations shown in the main manuscript. We also include the results on the family base level (corresponding to panels C and D of Figure 3 in the main paper).

BMI prediction from American Gut microbiome profiles

Finding consistent gut microbial signatures that are predictive of a person's body mass index (BMI) remains a non-trivial problem. Several early studies argued that obesity is associated with phylum-level changes in the microbiome [3], including increased Firmicutes to Bacteroidetes phyla ratios [4], often referred to as a hallmark predictor of obesity. The authors in [5] and [6] were among the first to identify a small set of microbial genera that were (moderately) predictive of host BMI using sparse log-contrast models on the COMBO microbiome dataset [7].

Using `trac`, we revisit BMI prediction from microbial abundance data using a subset of the American Gut Project (AGP) data comprising $p = 1387$ OTUs across $n = 6266$ participants in the lean to obese BMI range. The standard `trac` model ($a = 1$) with the 1SE rule identified a model with 132 predictors, consisting of aggregations across *all* taxonomic levels. Table 11 summarizes the 15 strongest predictors which include the kingdom Bacteria (vs. Archaea) as negative baseline, the phylum Bacteroidetes and several families and genera in the class Clostridia (which belongs to the Firmicutes phylum) with positive associations. The strongest positive OTU level predictor is an unknown species belonging to the Ruminococcaceae family. Figure 2 shows the corresponding `trac` model BMI predictions (with 1SE rule) vs. measured BMI on the test set (split 1). The out-of-sample test error on this split is 15.31, and roughly 16 on average across all ten splits (see Table 1). Standard `trac`, weighted `trac`, and sparse log-contrast models show similar performance in terms of test error ($16 - 17$) across all taxon base levels, with sparsity levels between 73 and 122 on OTU and genus level, and about 23-27 on the family level.

The standard `trac` model contains aggregations across all taxonomic levels. For instance, on the genus level, `trac` selects Blautia, Dorea, and Ruminococcus as positive predictors.

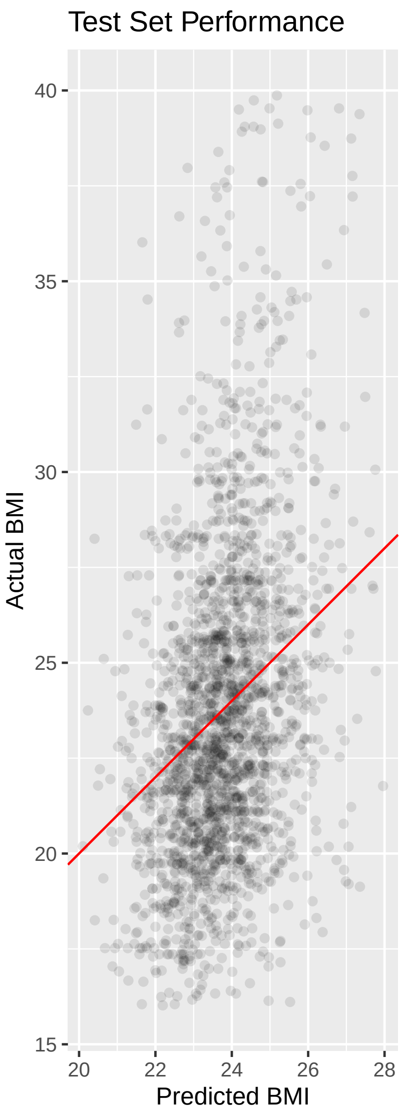

Figure 2: A scatter plot of measured BMI (y-axis) vs. `trac` model BMI predictions on a test set of $n = 2088$ AGP participants shows that predicted BMIs largely cover the "normal" BMI range between 20 and 28 with an overall test set correlation of 0.33. This model has 132 selected taxa, ranging from Kingdom to OTU levels. Table 11 shows the top 15 aggregations with largest α-coefficients.

**Base Level**	p	**trac (a = 1)**	**trac (a = 1/2)**	**Sparse Log-Contrast**
OTU	1387	16 (115)	16 (100)	16 (81)
Genus	824	16 (73)	16 (111)	16 (122)
Family	199	17 (27)	17 (23)	17 (24)

Table 1: Average out-of-sample test errors (model sparsity in parenthesis) for `trac` ($a = \{1, 1/2\}$) and sparse log-contrast models, respectively. Each row considers a different base level (OTU, genus, and family). Each number is averaged over ten different training/test splits of the Gut (AGP), BMI data.

The strongest overall positive predictors are the Bacteroidetes phylum, and the Ruminococcaceae, Lachnospiraceae, and Clostridiales families. The Lachnospiraceae/Bacteria ratio is also the first log-contrast to enter the `trac` aggregation path on the AGP data. The Erysipelotrichaceae and the Mogibacteriaceae families are the strongest negative predictors. Consistent with our model, Mogibacteriaceae were shown to be more abundant in lean individuals [8], and Erysipelotrichaceae were recently reported to be more abundant in normal compared to obese people or subjects with metabolic disorder [9]. However, the fact that standard `trac` could not identify a simple sparse predictive aggregation model for BMI suggests that more complex statistical models are required for predictive modeling, including adjustment for available covariates such as diet, sex, and overall life style.

Predicting Central Park pH and soil moisture from microbial communities

Here, we complement the microbiome-pH analysis from the main text with an investigation of the relationship between soil microbiome and gravimetric moisture (% water) measurements in Central Park. Since pH and moisture measurements are uncorrelated in the Central Park dataset, we also investigated the similarity between the predictive aggregations for pH and moisture.

Standard `trac` inferred a predictive model of moisture consisting of 23 taxonomic aggregations, including the phylum Proteobacteria and the classes Alpha- and Deltaproteobacteria as strong positive predictors, and the phyla Verrucomicrobia, Actinobacteria, and the order Sphingobacteriales as strong negative predictors (see Table 15). On the test data (split 1), the correlation between model predictions and measurements was 0.42. Compared to pH, the reduced predictive power is in agreement with [10]'s observation about the smaller influence of SMD compared to pH on microbial composition. Nonetheless, `trac`'s taxonomic groupings provide meaningful information about the taxonomic structure of soil microbiota along moisture gradients. For example, the model supports the positive association between Proteobacteria and moisture, as previously observed in a study along a vegetation gradient on the Loess Plateau in China [11], and the negative effect of moisture on the phylum Verrucomicrobia and the positive effect on Deltaproteobacteria in the Giessen free-air CO2 enrichment (Gi-FACE) experiment [12]. The Gi-FACE study, however, also reported several relationships between the microbiome and the soil moisture that are incongruent with our model, including the role of Acidobacteria.

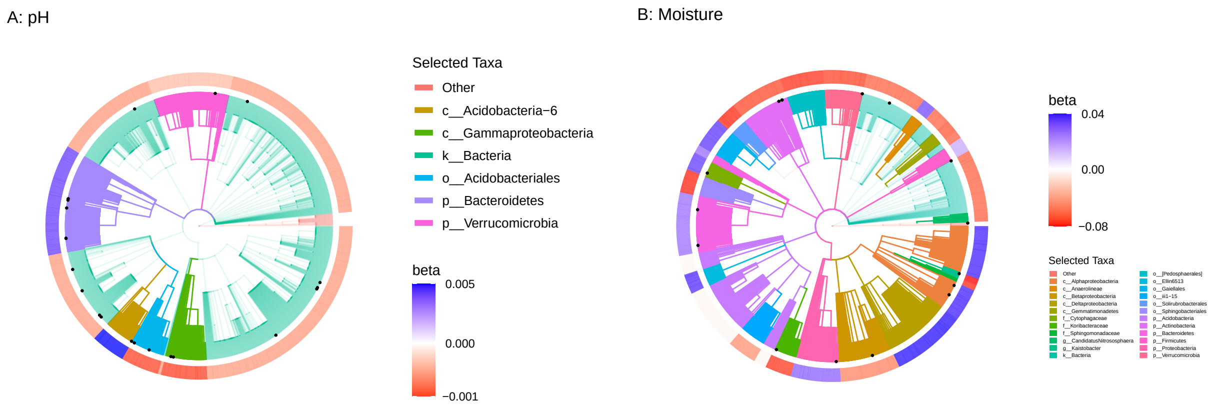

Figure 3: Taxonomic aggregations (as highlighted by branch colors) inferred by `trac` ($a = 1$), that are predictive of Central Park soil pH and moisture, respectively. The color coding on the outermost ring corresponds to the estimated leaf coefficients β and are in units of the response (which differs in the two cases).

Figure 3 compares the aggregations across the taxonomic tree that were found by standard `trac` for soil pH and moisture prediction, respectively. We observe that only the phyla Bacteroidetes and Verrucomicrobia, and the order Acidobacteriales are common in both models, confirming that the relevant taxonomic aggregations depend on the response

variable being predicted.

Finally, we observe similar prediction performance in terms of test error $(40 - 45)$, with standard `trac` being outperformed by the other methods across all base level aggregations. For moisture prediction, weighted `trac` provides an excellent trade-off between model interpretability and predictability.

**Base Level**	p	**trac (a = 1)**	**trac (a = 1/2)**	**Sparse Log-Contrast**
OTU	3379	42 (8)	40 (13)	40 (23)
Genus	2779	42 (5)	40 (17)	41 (19)
Family	1492	45 (4)	42 (12)	41 (16)

Table 2: Average out-of-sample test errors (model sparsity in parenthesis) for `trac` ($a = \{1, 1/2\}$) and sparse log-contrast models, respectively. Each row considers a different base level (OTU, genus, and family). Each number is averaged over ten different training/test splits of the Central Park soil, Moisture data.

Primary bacterial production in the Fram Strait

Current estimates suggest that the ocean microbiome could be responsible for about half of all primary production occurring on Earth [13, 14]. While net primary production is known to be highly influenced by a multitude of environmental drivers, including light, nutrients, and temperature [15], it is not yet established whether amplicon sequencing data alone contain enough information to serve as a stable predictor of (regional) marine primary production.

To investigate this relationship we consider a marine dataset, put forward in [16], that covers the Fram Strait, the main gateway between the North Atlantic and Arctic Oceans. The Fram Strait comprises two distinct oceanic regions, the northward flowing West Spitsbergen Current (WSC), and the East Greenland Current (EGC) flowing southward along the Greenland shelf. Recent ocean simulations, however, suggest substantial horizontal mixing and exchange by eddies between the two regions. We thus trained regression models from amplicon data across both regions and considered the available leucine incorporation (as proxy to bacterial production) as the outcome [16]. We learned separate models for the two different size fractions: $p = 4530$ free-living (FL) taxa in the $0.22\mu m$ fraction, and $p = 3320$ particle-associated (PA) taxa in $3\mu m$ fraction.

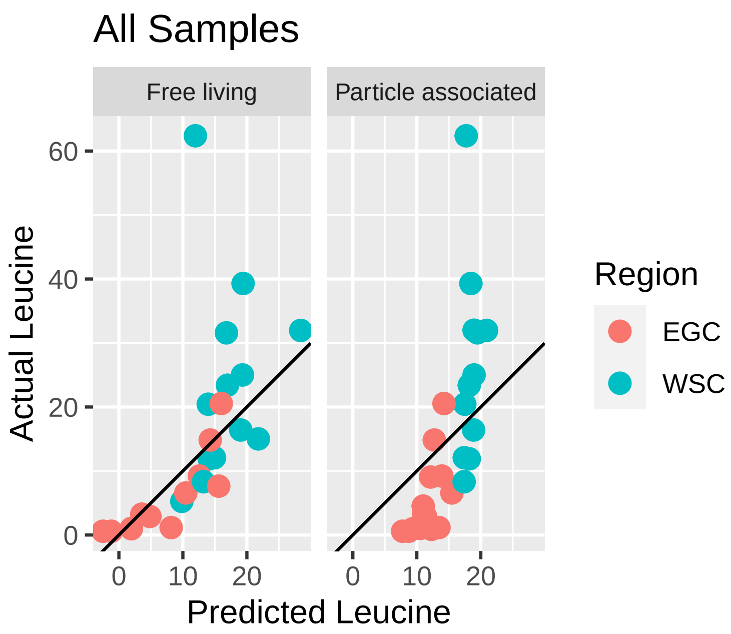

Figure 4: Predictions by `trac` ($a = 1$) of primary production (leucine) from free living (FL) and particle associated (PA) taxa. The data points are colored by region in the Fram Strait: West Spitsbergen Current (WSC), and the East Greenland Current (EGC). The correlation between predicted and measured leucine (on the test set of split 1) is 0.57 for FL taxa and 0.90 and PA taxa, respectively. Tables 20 and 23 show the selected taxa for these models.

On the FL dataset, `trac` ($a = 1$) identifies a parsimonious model, comprising three aggregated taxonomic groups, strongly associated with bacterial production. The two classes Gammaproteobacteria and Alphaproteobacteria are negatively associated, and the family Flavobacteriaceae is positively associated with bacterial production, leading to a two-factor

log-contrast model. On the PA dataset, standard `trac` infers a single predictive log-contrast with the Flavobacteriaceae family being positively associated and the entire phylum Proteobacteria negatively associated with primary production. On the test data (split 1), the PA model predictions show a correlation of 0.90 with the measurements. Figure 4 summarizes the scatter plots of leucine measurements vs. `trac` predictions for the two size fractions, colored by region WSC and EGC, respectively.

We observe that the PA model appears to serve as an implicit region classifier since predicted leucine values of < 17 belong uniquely to samples in the low-productivity EGC region (see top right panel in Figure 4). Our model suggests an important positive association of the heterotrophic Flavobacteriaceae with primary production, independent of size class. Flavobacteriaceae are known to strongly contribute to mineralization of primary-produced organic matter (see [17] and references therein), thus suggesting an indirect relationship between Flavobacteriaceae and primary production. However, previous studies in South polar front and antarctic zone postulated a strong role of Flavobacteriaceae for polar primary production [18].

As highlighted in Tables 3 and 4, weighted `trac` and log-contrast models lead to sparse models and outperform standard `trac` in terms of average test error. In the FL data set (data split 1), weighted `trac` selects both higher order aggregations and two OTUs both of which are also selected by the log-contrast models. For the PA dataset, all models result in single log-ratio models, either on the phylum/family level or OTU level, respectively.

**Base Level**	p	**trac (a = 1)**	**trac (a = 1/2)**	**Sparse Log-Contrast**
OTU	3320	1.3e+02 (4)	1.2e+02 (5)	84 (5)
Genus	1796	1.1e+02 (5)	1e+02 (4)	81 (4)
Family	597	1.2e+02 (3)	1e+02 (4)	99 (6)

Table 3: Average out-of-sample test errors (model sparsity in parenthesis) for `trac` ($a = \{1, 1/2\}$) and sparse log-contrast models, respectively. Each row considers a different base level (OTU, genus, and family). Each number is averaged over ten different training/test splits of the Fram Strait (PA) data.

**Base Level**	p	**trac (a = 1)**	**trac (a = 1/2)**	**Sparse Log-Contrast**
OTU	4510	1.9e+02 (2)	1.5e+02 (5)	1.7e+02 (4)
Genus	2930	1.9e+02 (3)	1.5e+02 (4)	1.4e+02 (6)
Family	1125	1.8e+02 (4)	1.4e+02 (4)	1.5e+02 (4)

Table 4: Average out-of-sample test errors (model sparsity in parenthesis) for `trac` ($a = \{1, 1/2\}$) and sparse log-contrast models, respectively. Each row considers a different base level (OTU, genus, and family). Each number is averaged over ten different training/test splits of the Fram Strait (FL) data.

Global predictive model of ocean salinity from Tara data

We complement the Tara data set analysis from the main text with showing the scatter plot of measured vs. predicted salinity for the standard trac model (trained on data split 1) in Figure 5.

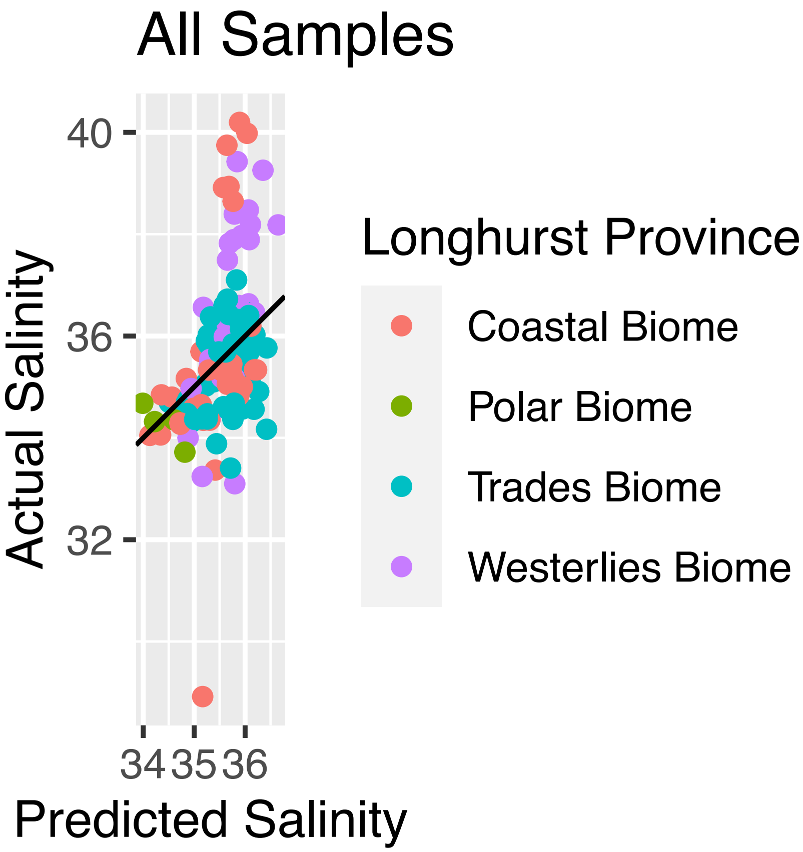

Figure 5: Measured salinity (y-axis) vs. standard trac ($a = 1$) model prediction (x-axis) on the Tara data (model training performed on data split 1). Each sample is colored by one of the four Longhurst Biome definitions. Outliers to the model are located in Coastal and Westerlies Biomes.

Table 5: Coefficients selected by `trac` (a = 1) for Gut (HIV): sCD14

Kingdom	Phylum	Class	Order	Family	Genus	Species	OTU	α
Bacteria	Firmicutes	Clostridia	Clostridiales	Ruminococcaceae				2221.75
Bacteria	Firmicutes	Clostridia	Clostridiales	Lachnospiraceae				-1644.86
Bacteria	Actinobacteria							-501.43
Bacteria								-362.27
Bacteria	Bacteroidetes	Bacteroidia	Bacteroidales	Bacteroidaceae	Bacteroides			286.80

Table 6: Coefficients selected by `trac` (a = 1/2) for Gut (HIV): sCD14

Kingdom	Phylum	Class	Order	Family	Genus	Species	OTU	α
Bacteria	Firmicutes	Clostridia	Clostridiales	Ruminococcaceae				629.10
Bacteria	Actinobacteria							-570.60
Bacteria	Firmicutes	Negativicutes	Selenomonadales	Veillonellaceae	Mitsuokella	-	Otu000070	-128.83
Bacteria	Firmicutes	Clostridia	Clostridiales	Lachnospiraceae	Lachnospira			-125.49
Bacteria	Firmicutes	Clostridia	Clostridiales	Ruminococcaceae	Subdoligranulum			121.80
Bacteria	Bacteroidetes	Bacteroidia	Bacteroidales	Bacteroidaceae	Bacteroides			82.62
Bacteria	Bacteroidetes	Bacteroidia	Bacteroidales	Bacteroidaceae	Bacteroides	-	Otu000014	51.81
Bacteria	Firmicutes	Clostridia	Clostridiales	Lachnospiraceae	Lachnospira	-	Otu000038	-49.69
Bacteria	Bacteroidetes	Bacteroidia	Bacteroidales	Prevotellaceae	Alloprevotella	-	Otu000011	41.31
Bacteria	Firmicutes	Clostridia	Clostridiales	Ruminococcaceae	Incertae_Sedis	-	Otu000073	-39.42
Bacteria	Actinobacteria	Actinobacteria	Bifidobacteriales	Bifidobacteriaceae	Bifidobacterium	-	Otu000098	-12.61

D Additional Selected Coefficient Tables

Table 7: Coefficients selected by the sparse log-contrast method for Gut (HIV): sCD14

Kingdom	Phylum	Class	Order	Family	Genus	Species	OTU	β
Bacteria	Bacteroidetes	Bacteroidia	Bacteroidales	Bacteroidaceae	Bacteroides	-	Otu000014	123.92
Bacteria	Firmicutes	Negativicutes	Selenomonadales	Veillonellaceae	Mitsuokella	-	Otu000070	-105.59
Bacteria	Firmicutes	Clostridia	Clostridiales	Ruminococcaceae	-	-	Otu000048	83.15
Bacteria	Firmicutes	Clostridia	Clostridiales	Lachnospiraceae	Lachnospira	-	Otu000038	-79.26
Bacteria	Actinobacteria	Coriobacteriia	Coriobacteriales	Coriobacteriaceae	Collinsella	-	Otu000230	-71.72
Bacteria	Bacteroidetes	Bacteroidia	Bacteroidales	Prevotellaceae	Alloprevotella	-	Otu000011	59.25
Bacteria	Actinobacteria	Actinobacteria	Bifidobacteriales	Bifidobacteriaceae	Bifidobacterium	-	Otu000098	-42.28
Bacteria	Firmicutes	Clostridia	Clostridiales	Ruminococcaceae	-	-	Otu000174	16.33
Bacteria	Proteobacteria	Deltaproteobacteria	Desulfovibrionales	Desulfovibrionaceae	Desulfovibrio	-	Otu000143	16.21

Table 8: Coefficients selected by `trac` on family level (a = 1) for Gut (HIV): sCD14

**Kingdom**	**Phylum**	**Class**	**Order**	**Family**	**Genus**	**Species**	**OTU**	α
Bacteria	Actinobacteria							-440.80
Bacteria								303.19
Bacteria	Cyanobacteria	Melainabacteria	Gastranaerophilales					137.61

Table 9: Coefficients selected by `trac` on family level (a = 1/2) for Gut (HIV): sCD14

**Kingdom**	**Phylum**	**Class**	**Order**	**Family**	**Genus**	**Species**	**OTU**	α
Bacteria	Actinobacteria							-419.10
Bacteria								301.98
Bacteria	Proteobacteria	Gammaproteobacteria	Enterobacteriales	Enterobacteriaceae				112.87
Bacteria	Bacteroidetes	Bacteroidia	Bacteroidales	Bacteroidaceae				9.42
Bacteria	Proteobacteria	Gammaproteobacteria	Aeromonadales	Succinivibrionaceae				-5.18

Table 10: Coefficients selected by the sparse log-contrast method on family level for Gut (HIV): sCD14

**Kingdom**	**Phylum**	**Class**	**Order**	**Family**	**Genus**	**Species**	**OTU**	β
Life	Bacteria	Actinobacteria	Coriobacteriia	Coriobacteriales	Coriobacteriaceae			-317.40
Life	Bacteria	Proteobacteria	Gammaproteobacteria	Enterobacteriales	Enterobacteriaceae			177.47
Life	Bacteria	Cyanobacteria	Melainabacteria	Gastranaerophilales	[Unclassified]			138.24
Life	Bacteria	Actinobacteria	Actinobacteria	Bifidobacteriales	Bifidobacteriaceae			-68.26
Life	Bacteria	Firmicutes	Clostridia	Clostridiales	Defluviitaleaceae			44.07
Life	Bacteria	Proteobacteria	Alphaproteobacteria	[Unclassified]	[Unclassified]			25.88

Table 11: Top 15 coefficients selected by `trac` (a = 1) for Gut (AGP): BMI

**Kingdom**	**Phylum**	**Class**	**Order**	**Family**	**Genus**	**Species**	**OTU**	α
Bacteria								-11.95
Bacteria	Firmicutes	Clostridia	Clostridiales	Ruminococcaceae				2.86
Bacteria	Firmicutes	Clostridia	Clostridiales	Lachnospiraceae				2.23
Bacteria	Bacteroidetes							1.45
Bacteria	Firmicutes	Clostridia	Clostridiales					1.18
Bacteria	Proteobacteria	Gammaproteobacteria	Enterobacteriales	Enterobacteriaceae				0.90
Bacteria	Firmicutes	Erysipelotrichi	Erysipelotrichales	Erysipelotrichaceae				-0.80
Bacteria	Firmicutes	Clostridia	Clostridiales	Lachnospiraceae	Blautia			0.73
Bacteria	Firmicutes	Bacilli	Lactobacillales					0.72
Bacteria	Firmicutes	Clostridia	Clostridiales	Veillonellaceae				0.71
Bacteria	Firmicutes	Clostridia	Clostridiales	Lachnospiraceae	Dorea			0.51
Bacteria	Firmicutes	Clostridia	Clostridiales	Ruminococcaceae	Ruminococcus			0.49
Bacteria	Firmicutes	Clostridia	Clostridiales	[Mogibacteriaceae]				-0.36
Bacteria	Bacteroidetes	Bacteroidia	Bacteroidales	[Barnesiellaceae]				0.32
Bacteria	Firmicutes	Clostridia	Clostridiales	Ruminococcaceae	-	-	4356062	0.30

Table 12: Top 15 coefficients selected by `trac` (a = 1/2) for Gut (AGP): BMI

Kingdom	Phylum	Class	Order	Family	Genus	Species	OTU	α
Bacteria	Firmicutes	Erysipelotrichi	Erysipelotrichales	Erysipelotrichaceae				-0.30
Bacteria	Firmicutes	Clostridia	Clostridiales	Ruminococcaceae	-	-	4356062	0.28
Bacteria	Proteobacteria	Gammaproteobacteria	Enterobacteriales	Enterobacteriaceae				-0.24
Bacteria	Firmicutes	Bacilli	Lactobacillales					0.23
Bacteria	Proteobacteria	Gammaproteobacteria	Pasteurellales	Pasteurellaceae	Haemophilus	parainfluenzae	4477696	-0.21
Bacteria	Firmicutes	Clostridia	Clostridiales	Lachnospiraceae	Dorea	-	181871	0.19
Bacteria	Firmicutes	Clostridia	Clostridiales	Lachnospiraceae	Blautia	-	4361189	0.19
Bacteria	Firmicutes	Clostridia	Clostridiales	[Tissierellaceae]	Finegoldia	-	1096610	0.17
Bacteria	Firmicutes	Erysipelotrichi	Erysipelotrichales	Erysipelotrichaceae	Catenibacterium	-	4480861	0.16
Bacteria	Actinobacteria	Actinobacteria						-0.15
Bacteria	Firmicutes	Erysipelotrichi	Erysipelotrichales	Erysipelotrichaceae	-	-	145801	-0.14
Bacteria	Firmicutes	Clostridia	Clostridiales	-	-	-	195004	0.14
Bacteria	Bacteroidetes	Bacteroidia	Bacteroidales	[Barnesiellaceae]				0.13
Bacteria	Firmicutes	Bacilli	Bacillales	Staphylococcaceae	Staphylococcus	aureus	4446058	-0.13
Bacteria	Actinobacteria	Coriobacteriia	Coriobacteriales	Coriobacteriaceae	Eggerthella	lenta	4393532	-0.12

Table 13: Top 15 coefficients selected by the sparse log-contrast method for Gut (AGP): BMI

Kingdom	Phylum	Class	Order	Family	Genus	Species	OTU	β
Bacteria	Firmicutes	Clostridia	Clostridiales	Ruminococcaceae	-	-	4356062	0.29
Bacteria	Proteobacteria	Gammaproteobacteria	Pasteurellales	Pasteurellaceae	Haemophilus	parainfluenzae	4477696	-0.19
Bacteria	Firmicutes	Erysipelotrichi	Erysipelotrichales	Erysipelotrichaceae	-	-	145801	-0.16
Bacteria	Firmicutes	Clostridia	Clostridiales	Lachnospiraceae	Blautia	-	4361189	0.16
Bacteria	Firmicutes	Clostridia	Clostridiales	Lachnospiraceae	Dorea	-	181871	0.15
Bacteria	Firmicutes	Erysipelotrichi	Erysipelotrichales	Erysipelotrichaceae	Catenibacterium	-	4480861	0.14
Bacteria	Firmicutes	Clostridia	Clostridiales	[Tissierellaceae]	Finegoldia	-	1096610	0.13
Bacteria	Firmicutes	Bacilli	Bacillales	Staphylococcaceae	Staphylococcus	aureus	4446058	-0.11
Bacteria	Firmicutes	Clostridia	Clostridiales	Lachnospiraceae	-	-	4457438	0.11
Bacteria	Firmicutes	Clostridia	Clostridiales	Ruminococcaceae	-	-	2018038	0.11
Bacteria	Actinobacteria	Coriobacteriia	Coriobacteriales	Coriobacteriaceae	Eggerthella	lenta	4393532	-0.10
Bacteria	Firmicutes	Erysipelotrichi	Erysipelotrichales	Erysipelotrichaceae	Clostridium	saccharogumia	4379449	-0.10
Bacteria	Firmicutes	Clostridia	Clostridiales	-	-	-	340876	-0.10
Bacteria	Firmicutes	Clostridia	Clostridiales	-	-	-	173876	-0.09
Bacteria	Proteobacteria	Betaproteobacteria	Burkholderiales	Oxalobacteraceae	Oxalobacter	formigenes	7366	-0.09

Table 14: Coefficients selected by `trac` (a = 1) for Central Park Soil: pH

Kingdom	Phylum	Class	Order	Family	Genus	Species	OTU	α
Bacteria								-0.74
Bacteria	Acidobacteria	Acidobacteria-6						0.58
Bacteria	Bacteroidetes							0.45
Bacteria	Proteobacteria	Gammaproteobacteria						-0.19
Bacteria	Acidobacteria	Acidobacteriia	Acidobacteriales					-0.13
Bacteria	Verrucomicrobia							0.03

Table 15: Top 15 coefficients selected by `trac` (a = 1/2) for Central Park Soil: pH

**Kingdom**	**Phylum**	**Class**	**Order**	**Family**	**Genus**	**Species**	**OTU**	α
Bacteria	Acidobacteria	Acidobacteria-6						0.38
Bacteria	Proteobacteria	Gammaproteobacteria	Xanthomonadales	Xanthomonadaceae				-0.23
Bacteria	WPS-2							-0.19
Bacteria	Gemmatimonadetes	Gemm-1						-0.11
Bacteria	Bacteroidetes	Cytophagia	Cytophagales	Cytophagaceae				0.09
Bacteria	Proteobacteria	Gammaproteobacteria	Xanthomonadales	Xanthomonadaceae	Rhodanobacter			-0.05
Bacteria	Bacteroidetes							0.05
Bacteria	Acidobacteria	Acidobacteria-6	iii1-15	RB40	-	-	OTU_444	0.05
Bacteria	Bacteroidetes	[Saprospirae]	[Saprospirales]	Chitinophagaceae	-	-	OTU_77	0.04
Bacteria	Proteobacteria	Alphaproteobacteria	Ellin329					-0.04
Bacteria	Acidobacteria	DA052	Ellin6513					-0.04
Bacteria	Bacteroidetes	[Saprospirae]	[Saprospirales]	Saprospiraceae				0.04
Bacteria	Bacteroidetes	Cytophagia	Cytophagales	Cytophagaceae	-	-	OTU_176	-0.04
Bacteria	Proteobacteria	Gammaproteobacteria	Alteromonadales	OM60				0.02
Bacteria	Chloroflexi	Ktedonobacteria	Ktedonobacterales	Ktedonobacteraceae				0.02

Table 16: Top 15 coefficients selected by the sparse log-contrast method for Central Park Soil: pH

**Kingdom**	**Phylum**	**Class**	**Order**	**Family**	**Genus**	**Species**	**OTU**	β
Bacteria	Bacteroidetes	[Saprospirae]	[Saprospirales]	Chitinophagaceae	-	-	OTU_77	0.08
Bacteria	Acidobacteria	Solibacteres	Solibacterales	Solibacteraceae	Candidatus Solibacter	-	OTU_114	-0.06
Bacteria	Acidobacteria	Acidobacteria-6	iii1-15	RB40	-	-	OTU_444	0.05
Bacteria	Acidobacteria	[Chloracidobacteria]	RB41	-	-	-	OTU_129299	0.04
Bacteria	Bacteroidetes	[Saprospirae]	[Saprospirales]	Chitinophagaceae	-	-	OTU_124173	-0.04
Bacteria	Actinobacteria	Actinobacteria	Actinomycetales	-	-	-	OTU_7	-0.04
Bacteria	Verrucomicrobia	[Spartobacteria]	[Chthoniobacterales]	[Chthoniobacteraceae]	-	-	OTU_335	0.03
Bacteria	Acidobacteria	Solibacteres	Solibacterales	Solibacteraceae	-	-	OTU_178	-0.02
Bacteria	Acidobacteria	DA052	Ellin6513	-	-	-	OTU_432	-0.02
Bacteria	Bacteroidetes	[Saprospirae]	[Saprospirales]	Saprospiraceae	-	-	OTU_77144	0.02
Bacteria	Proteobacteria	Deltaproteobacteria	Syntrophobacterales	Syntrophobacteraceae	-	-	OTU_407	-0.01
Bacteria	Proteobacteria	Gammaproteobacteria	Enterobacteriales	Enterobacteriaceae	Klebsiella	-	OTU_62	-0.01
Bacteria	Planctomycetes	Planctomycetia	Pirellulales	Pirellulaceae	-	-	OTU_12778	-0.01
Bacteria	Proteobacteria	Alphaproteobacteria	Ellin329	-	-	-	OTU_80	-0.01
Bacteria	Bacteroidetes	[Saprospirae]	[Saprospirales]	Chitinophagaceae	-	-	OTU_190	-0.01

Table 17: Top 15 coefficients selected by `trac` (a = 1) for Central Park Soil: Mois

**Kingdom**	**Phylum**	**Class**	**Order**	**Family**	**Genus**	**Species**	**OTU**	α
Bacteria								-26.58
Bacteria	Proteobacteria							13.68
Bacteria	Proteobacteria	Deltaproteobacteria						9.71
Bacteria	Proteobacteria	Alphaproteobacteria						6.77
Bacteria	Bacteroidetes							4.89
Bacteria	Acidobacteria							4.68
Bacteria	Bacteroidetes	Sphingobacteriia	Sphingobacteriales					-3.25
Bacteria	Actinobacteria	Thermoleophilia	Gaiellales					3.01
Bacteria	Verrucomicrobia							-2.66
Bacteria	Proteobacteria	Alphaproteobacteria	Sphingomonadales	Sphingomonadaceae				-2.40
Bacteria	Actinobacteria							-2.38
Bacteria	Proteobacteria	Betaproteobacteria						-2.16
Bacteria	Acidobacteria	Acidobacteriia	Acidobacteriales	Koribacteraceae				-1.99
Bacteria	Verrucomicrobia	[Pedosphaerae]	[Pedosphaerales]					-1.37
Bacteria	Actinobacteria	Thermoleophilia	Solirubrobacterales					-1.24

Table 18: Top 15 coefficients selected by `trac` (a = 1/2) for Central Park Soil: Mois

Kingdom	Phylum	Class	Order	Family	Genus	Species	OTU	α
Bacteria	Proteobacteria							6.07
Bacteria	Verrucomicrobia							-3.44
Bacteria	Proteobacteria	Alphaproteobacteria	Sphingomonadales	Sphingomonadaceae	Kaistobacter			-2.02
Bacteria	Proteobacteria	Deltaproteobacteria						1.83
Bacteria	Actinobacteria							-1.51
Bacteria	Actinobacteria	Thermoleophilia	Solirubrobacterales					-0.71
Bacteria	Actinobacteria	Thermoleophilia	Solirubrobacterales	Conexibacteraceae				-0.57
Bacteria	Acidobacteria	Acidobacteriia	Acidobacteriales	Koribacteraceae	-	-	OTU_132332	-0.47
Archaea	Crenarchaeota	Thaumarchaeota	Nitrososphaerales	Nitrososphaeraceae	CandidatusNitrososphaera			-0.30
Bacteria	Proteobacteria	Alphaproteobacteria	Ellin329	-	-	-	OTU_2107	0.24
Bacteria	Proteobacteria	Alphaproteobacteria	Rickettsiales	mitochondria	-	-	OTU_504	0.17
Archaea	Crenarchaeota	Thaumarchaeota	Cenarchaeales	SAGMA-X				0.14
Bacteria	Proteobacteria	Alphaproteobacteria	Rhizobiales	Hyphomicrobiaceae	Hyphomicrobium			0.14
Bacteria	Proteobacteria	Deltaproteobacteria	Desulfuromonadales	Geobacteraceae	Geobacter			0.12
Bacteria	Bacteroidetes	[Saprospirae]	[Saprospirales]	Chitinophagaceae	-	-	OTU_4903	0.09

Table 19: Coefficients selected by the sparse log-contrast method for Central Park Soil: Mois

Kingdom	Phylum	Class	Order	Family	Genus	Species	OTU	β
Bacteria	Acidobacteria	Acidobacteriia	Acidobacteriales	Koribacteraceae	-	-	OTU_132332	-0.77
Bacteria	Bacteroidetes	Cytophagia	Cytophagales	Cytophagaceae	-	-	OTU_103638	0.46
Bacteria	Bacteroidetes	[Saprospirae]	[Saprospirales]	Chitinophagaceae	-	-	OTU_4903	0.33
Bacteria	Proteobacteria	Betaproteobacteria	MND1	-	-	-	OTU_811	0.27
Bacteria	Actinobacteria	Acidimicrobiia	Acidimicrobiales	-	-	-	OTU_461	-0.20
Bacteria	Proteobacteria	Gammaproteobacteria	Xanthomonadales	Sinobacteraceae	-	-	OTU_1132	0.20
Bacteria	Planctomycetes	Phycisphaerae	WD2101	-	-	-	OTU_132692	-0.16
Bacteria	Proteobacteria	Alphaproteobacteria	Ellin329	-	-	-	OTU_2107	0.13
Bacteria	Bacteroidetes	[Saprospirae]	[Saprospirales]	Chitinophagaceae	-	-	OTU_91357	-0.12
Bacteria	Proteobacteria	Alphaproteobacteria	Rickettsiales	mitochondria	-	-	OTU_504	0.11
Bacteria	Actinobacteria	Acidimicrobiia	Acidimicrobiales	-	-	-	OTU_669	-0.11
Bacteria	Proteobacteria	Alphaproteobacteria	Sphingomonadales	Sphingomonadaceae	Kaistobacter	-	OTU_10329	-0.09
Bacteria	Actinobacteria	Acidimicrobiia	Acidimicrobiales	-	-	-	OTU_1582	-0.08
Bacteria	Verrucomicrobia	-	-	-	-	-	OTU_1207	0.03
Archaea	Crenarchaeota	Thaumarchaeota	Cenarchaeales	SAGMA-X	-	-	OTU_208	0.01

Table 20: Coefficients selected by `trac` (a = 1) for Fram Strait (FL): Leucine

Kingdom	Phylum	Class	Order	Family	Genus	Species	OTU	α
Bacteria	Bacteroidetes	Flavobacteriia	Flavobacteriales	Flavobacteriaceae				27.90
Bacteria	Proteobacteria	Alphaproteobacteria						-23.40
Bacteria	Proteobacteria	Gammaproteobacteria						-4.49

Table 21: Coefficients selected by `trac` (a = 1/2) for Fram Strait (FL): Leucine

Kingdom	Phylum	Class	Order	Family	Genus	Species	OTU	α
Bacteria	Bacteroidetes	Flavobacteriia	Flavobacteriales	Flavobacteriaceae				14.30
Bacteria	Proteobacteria	Alphaproteobacteria	Rhodospirillales					-8.99
Bacteria	Marinimicrobia(SAR406clade)							-4.30
Bacteria	Bacteroidetes	Flavobacteriia	Flavobacteriales	NS9 marine group	-	-	otu117	-0.79
Bacteria	Proteobacteria	Deltaproteobacteria	SAR324 clade(Marine group B)	-	-	-	otu14	-0.22

Table 22: Coefficients selected by the sparse log-contrast method for Fram Strait (FL): Leucine

Kingdom	Phylum	Class	Order	Family	Genus	Species	OTU	β
Bacteria	Bacteroidetes	Flavobacteriia	Flavobacteriales	Flavobacteriaceae	Ulvibacter	-	otu9	1.44
Bacteria	Bacteroidetes	Flavobacteriia	Flavobacteriales	NS9 marine group	-	-	otu117	-0.83
Bacteria	Proteobacteria	Deltaproteobacteria	SAR324 clade(Marine group B)	-	-	-	otu14	-0.61

Table 23: Coefficients selected by `trac` (a = 1) for Fram Strait (PA): Leucine

Kingdom	Phylum	Class	Order	Family	Genus	Species	OTU	α
Bacteria	Proteobacteria							-13.93
Bacteria	Bacteroidetes	Flavobacteriia	Flavobacteriales	Flavobacteriaceae				13.93

Table 24: Coefficients selected by `trac` (a = 1/2) for Fram Strait (PA): Leucine

Kingdom	Phylum	Class	Order	Family	Genus	Species	OTU	α
Bacteria	Planctomycetes							-6.79
Bacteria	Proteobacteria	Alphaproteobacteria	Rhodobacterales	Rhodobacteraceae				6.79

Table 25: Coefficients selected by the sparse log-contrast method for Fram Strait (PA): Leucine

Kingdom	Phylum	Class	Order	Family	Genus	Species	OTU	β
Bacteria	Proteobacteria	Alphaproteobacteria	Rhodobacterales	Rhodobacteraceae	Sulfitobacter	-	otu11	1.41
Bacteria	Proteobacteria	Deltaproteobacteria	Bdellovibrionales	Bdellovibrionaceae	OM27 clade	-	otu93	-1.41

Table 26: Coefficients selected by `trac` (a = 1) for Ocean (TARA): Salinity

Kingdom	Phylum	Class	Order	Family	Genus	Species	OTU	α
Bacteria	Proteobacteria	Alphaproteobacteria						4.00
Bacteria								-2.92
Bacteria	Bacteroidetes							-1.38
Bacteria	Proteobacteria	Gammaproteobacteria						0.30

Table 27: Coefficients selected by `trac` (a = 1/2) for Ocean (TARA): Salinity

Kingdom	Phylum	Class	Order	Family	Genus	Species	OTU	α
Bacteria	Bacteroidetes	Flavobacteria	Flavobacteriales	NS9marinegroup				-0.96
Bacteria	Proteobacteria	Alphaproteobacteria	SAR11clade					0.55
Bacteria	Proteobacteria	Alphaproteobacteria						0.38
Bacteria	Proteobacteria	Gammaproteobacteria	Oceanospirillales	Halomonadaceae				-0.37
Bacteria	Cyanobacteria							0.25
Bacteria	Cyanobacteria	Cyanobacteria	SubsectionI	FamilyI	Synechococcus			0.12
Bacteria	Proteobacteria	Gammaproteobacteria	E01-9C-26 marine group	-	-	JF747664.1.1516	OTU520	0.09
Bacteria	Proteobacteria	Gammaproteobacteria	Alteromonadales	Alteromonadaceae	Marinobacter			-0.07
Bacteria	Cyanobacteria	Cyanobacteria						0.02
Bacteria	Bacteroidetes							-0.02

Table 28: Coefficients selected by the sparse log-contrast method for Ocean (TARA): Salinity

Kingdom	Phylum	Class	Order	Family	Genus	Species	OTU	β
Bacteria	Proteobacteria	Gammaproteobacteria	E01-9C-26 marine group	-	-	JF747664.1.1516	OTU520	0.25
Bacteria	Proteobacteria	Gammaproteobacteria	Oceanospirillales	JL-ETNP-Y6	-	GQ347814.1.1378	OTU925	-0.11
Bacteria	Proteobacteria	Gammaproteobacteria	Oceanospirillales	SAR86 clade	-	AACY020549891.3846.5359	OTU19	0.06
Bacteria	Verrucomicrobia	Verrucomicrobiae	Verrucomicrobiales	Verrucomicrobiaceae	Roseibacillus	GU062019.1.1504	OTU729	-0.05
Bacteria	Proteobacteria	Gammaproteobacteria	Alteromonadales	Alteromonadaceae	Melitea	HQ326447.1.1497	OTU2376	-0.05
Bacteria	Proteobacteria	Gammaproteobacteria	Salinisphaerales	Salinisphaeraceae	Salinisphaera	AB735546.1.1462	OTU1096	-0.04
Bacteria	Bacteroidetes	Flavobacteria	Flavobacteriales	NS9 marine group	-	HQ673682.1.1487	OTU1168	-0.04
Bacteria	Proteobacteria	Gammaproteobacteria	Alteromonadales	Idiomarinaceae	Idiomarina	EU440983.1.1508	OTU2517	-0.02
Bacteria	Actinobacteria	Acidimicrobiia	Acidimicrobiales	OCS155 marine group	-	AACY020396101.1882.3388	OTU56	0.01

References

[1] Léo Simpson, Patrick L. Combettes, and Christian L Müller. c-lasso - a Python package for constrained sparse and robust regression and classification. *Journal of Open Source Software*, 6(57):2844, 2021.

[2] Xiaohan Yan and Jacob Bien. Rare feature selection in high dimensions. *Journal of the American Statistical Association*, 0(just-accepted):1–30, 2020.

[3] P. J. Turnbaugh, M. Hamady, T. Yatsunenko, B. L. Cantarel, A. Duncan, R. E. Ley, M. L. Sogin, W. J. Jones, B. A. Roe, J. P. Affourtit, M. Egholm, B. Henrissat, A. C. Heath, R. Knight, and J. I. Gordon. A core gut microbiome in obese and lean twins. *Nature*, 457(7228):480–484, Jan 2009.

[4] Ruth E. Ley, Peter J. Turnbaugh, Samuel Klein, and Jeffrey I. Gordon. Microbial ecology: Human gut microbes associated with obesity. *Nature*, 2006.

[5] Wei Lin, Pixu Shi, Rui Feng, and Hongzhe Li. Variable selection in regression with compositional covariates. *Biometrika*, 101:785–797, 11 2014.

[6] Pixu Shi, Anru Zhang, and Hongzhe Li. Regression analysis for microbiome compositional data. *Ann. Appl. Stat.*, 10(2):1019–1040, 06 2016.

[7] G. D. Wu, J. Chen, C. Hoffmann, K. Bittinger, Y.-Y. Chen, S. A. Keilbaugh, M. Bewtra, D. Knights, W. A. Walters, R. Knight, R. Sinha, E. Gilroy, K. Gupta, R. Baldassano, L. Nessel, H. Li, F. D. Bushman, and J. D. Lewis. Linking Long-Term Dietary Patterns with Gut Microbial Enterotypes. *Science*, 334(6052):105–108, 2011.

[8] Kaihei Oki, Mutsumi Toyama, Taihei Banno, Osamu Chonan, Yoshimi Benno, and Koichi Watanabe. Comprehensive analysis of the fecal microbiota of healthy Japanese adults reveals a new bacterial lineage associated with a phenotype characterized by a high frequency of bowel movements and a lean body type. *BMC Microbiology*, pages 5–11, 2016.

[9] Alejandra Chávez-Carbajal, Khemlal Nirmalkar, Ana Pérez-Lizaur, Fernando Hernández-Quiroz, Silvia Ramírez-Del-Alto, Jaime García-Mena, and César Hernández-Guerrero. Gut microbiota and predicted metabolic pathways in a sample of Mexican women affected by obesity and obesity plus metabolic syndrome. *International Journal of Molecular Sciences*, 20(2):1–18, 2019.

[10] Noah Fierer and Robert B Jackson. The diversity and biogeography of soil bacterial communities. *PNAS*, 103(3), 2006.

[11] Quanchao Zeng, Yanghong Dong, and Shaoshan An. Bacterial community responses to soils along a latitudinal and vegetation gradient on the Loess Plateau, China. *PLoS ONE*, 11(4):1–17, 2016.

[12] Alexandre B. de Menezes, Christoph Müller, Nicholas Clipson, and Evelyn Doyle. The soil microbiome at the Gi-FACE experiment responds to a moisture gradient but not to CO2 enrichment. *Microbiology (United Kingdom)*, 162(9):1572–1582, 2016.

[13] Alan Longhurst, Shubha Sathyendranath, Trevor Platt, and Carla Caverhill. An estimate of global primary production in the ocean from satellite radiometer data. *Journal of Plankton Research*, 17(6):1245–1271, 1995.

[14] Mary Ann Moran. The global ocean microbiome. *Science*, 350(6266), 2015.

[15] P W Boyd, S Sundby, and H.-O. Pörtner. Net primary production in the ocean. *Climate Change 2014: Impacts, Adaptation, and Vulnerability. Part A: Global and Sectoral Aspects. Contribution of Working Group II to the Fifth Assessment Report of the Intergovernmental Panel on Climate Change*, pages 133–136, 2014.

[16] Eduard Fadeev, Ian Salter, Vibe Schourup-Kristensen, Eva Maria Nöthig, Katja Metfies, Anja Engel, Judith Piontek, Antje Boetius, and Christina Bienhold. Microbial communities in the east and west fram strait during sea ice melting season. *Frontiers in Marine Science*, 5(NOV):1–21, 2018.

[17] John P. Bowman and David S. Nichols. Novel members of the family Flavobacteriaceae from Antarctic maritime habitats including Subsaximicrobium wynnwilliamsii gen. nov., sp. nov., Subsaximicrobium saxinquilinus sp. nov., Subsaxibacter broadyi gen. nov., sp. nov., Lacinutrix copepodicola gen. nov., sp. nov., and novel species of the genera Bizionia, Gelidibacter and Gillisia. *International Journal of Systematic and Evolutionary Microbiology*, 55(4):1471–1486, 2005.

[18] Guy C.J. Abell and John P. Bowman. Ecological and biogeographic relationships of class Flavobacteria in the Southern Ocean. *FEMS Microbiology Ecology*, 51(2):265–277, 2005.
